# Puma, noxa, p53, and p63 differentially mediate stress pathway induced apoptosis

**DOI:** 10.1038/s41419-021-03902-6

**Published:** 2021-06-30

**Authors:** Jun Wang, Holly R. Thomas, Zhang Li, Nan Cher (Florence) Yeo, Hannah E. Scott, Nghi Dang, Mohammed Iqbal Hossain, Shaida A. Andrabi, John M. Parant

**Affiliations:** 1 Department of Pharmacology and Toxicology, University of Alabama at Birmingham School of Medicine, Birmingham, AL USA; 2 Department of Cell, Developmental and Integrative Biology, University of Alabama at Birmingham, Birmingham, AL USA; 3 Department of Biology, University of Alabama at Birmingham Collage of Arts and Sciences Department and Genetics Department, University of Alabama at Birmingham School of Medicine, Birmingham, AL USA; 4 Department of Neurology, University of Alabama at Birmingham School of Medicine, Birmingham, AL USA

**Keywords:** Cancer genetics, Tumour-suppressor proteins, Apoptosis, Mechanisms of disease

## Abstract

Cellular stress can lead to several human disease pathologies due to aberrant cell death. The p53 family (tp53, tp63, and tp73) and downstream transcriptional apoptotic target genes (PUMA/BBC3 and NOXA/PMAIP1) have been implicated as mediators of stress signals. To evaluate the importance of key stress response components in vivo, we have generated zebrafish null alleles in puma, noxa, p53, p63, and p73. Utilizing these genetic mutants, we have deciphered that the apoptotic response to genotoxic stress requires p53 and puma, but not p63, p73, or noxa. We also identified a delayed secondary wave of genotoxic stress-induced apoptosis that is p53/puma independent. Contrary to genotoxic stress, ER stress-induced apoptosis requires p63 and puma, but not p53, p73, or noxa. Lastly, the oxidative stress-induced apoptotic response requires p63, and both noxa and puma. Our data also indicate that while the neural tube is poised for apoptosis due to genotoxic stress, the epidermis is poised for apoptosis due to ER and oxidative stress. These data indicate there are convergent as well as unique molecular pathways involved in the different stress responses. The commonality of puma in these stress pathways, and the lack of gross or tumorigenic phenotypes with puma loss suggest that a inhibitor of Puma may have therapeutic application. In addition, we have also generated a knockout of the negative regulator of p53, mdm2 to further evaluate the p53-induced apoptosis. Our data indicate that the p53 null allele completely rescues the mdm2 null lethality, while the puma null completely rescues the mdm2 null apoptosis but only partially rescues the phenotype. Indicating Puma is the key mediator of p53-dependent apoptosis. Interestingly the p53 homozygous null zebrafish develop tumors faster than the previously described p53 homozygous missense mutant zebrafish, suggesting the missense allele may be hypomorphic allele.

## Introduction

Cellular stress response occurs when homeostasis is perturbed [1]. Prolonged acute stress or chromic stress often results in cell death to remove the stressed cell from the organism. The apoptotic response to stress is often pathological and associated with human diseases [2–8]. Among stress pathways, DNA damage stress, unfolded protein stress, and oxidative stress responses have been linked to multiple human pathologies and can be distinguished by distinct proximal signaling components but can also converge downstream on the p53 family of stress sensors and the apoptotic signaling network [9–17]. Interestingly, the cellular response to a stress can also be cell/tissues dependent [18–23]. Deeper understanding of consequences of cellular stress and mediators of stress pathways in vivo will facilitate avenues to mediate disease pathogenesis.

The p53 family (tp53, tp63, and tp73) acts as mediators of apoptotic stress response [24–27]. The tumor suppressor p53 is activated by a number of cellular stressed including but not limited to genotoxic stress, ribosomal stress, and oncogenic stress [28–34]. Further, p63 and p73 have been shown to be required for doxorubicin-induced neural cell death in mouse embryos [35]; however not in irradiated mouse thymocytes [36]. This suggests tissue-specific influences occur. In addition, the transcription factor p63 can regulate the intrinsic apoptosis in response to ER stress through mediating Puma expression [19, 37, 38]. p73 can also mediate ROS stress to increase the BAK/BCL-2 ratio in human cells [39]. These studies suggest not only p53, but also p63 and p73 have the potential to mediate multiple stress-induced apoptotic outcomes through the induction of the pro-apoptotic Bcl-2 family members, such as PUMA and NOXA [40–42]. However, which of the p53 family and/or proapoptotic family are essential for the different stress responses is unclear.

Zebrafish is a useful model to understand the in vivo pathology associated with human diseases. We and others have demonstrated zebrafish are a model of cancer predisposition, heart development, neurodegeneration, and many others [43–47]. Many of diseases and stress pathways are conserved and ~82% of human disease genes have zebrafish homologs [48]. For example, in the p53 pathway p53, p63, and p73; the negative regulators mdm2 and mdm4; the downstream transcriptional targets, such as p21, puma, noxa, cyclin G, and gadd45a are conserved in 1:1 orthology. Here, we took advantage of the properties of zebrafish embryos to analyze apoptotic outcomes in response to genotoxic stress, ER stress, and oxidative stress in a number of genetic null animals. We generated six knockout alleles including puma/bbc3, noxa/pmaip1, p53, mdm2, p63, and p73 with multiple genome-editing techniques. Utilizing these mutants, we defined: (1) that the apoptosis response to genotoxic stress requires p53 and puma, but not p63, p73, or noxa. (2) The ER stress-induced apoptosis requires p63 and puma, but not p53, p73, or noxa. And (3) the oxidative stress-induced apoptotic response requires p63, and both noxa and puma. These data indicate there are convergent as well as unique molecular pathways involved in the different stress responses.

## Results

### Multiple cellular stresses induce transcriptional induction of puma and noxa in zebrafish

**Fig. 1:**
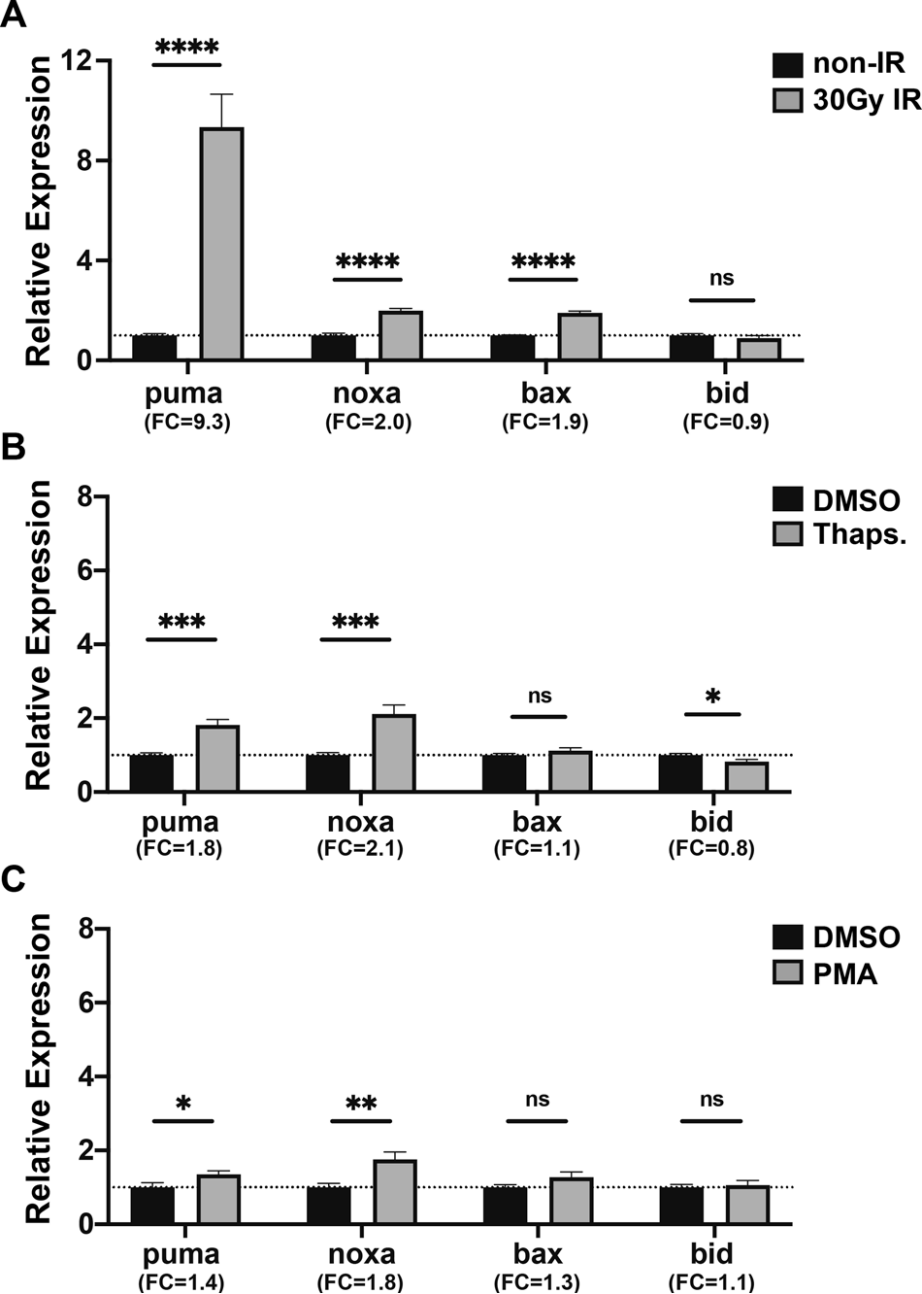
Quantitative real-time PCR (qRT-PCR) analysis of pro-apoptotic markers after IR- and drug-induction in wild-type zebrafish embryos. 24 hpf zebrafish embryos were treated with **A** 30 Gy IR-irradiation, **B** 5 μM Thapsigargin (Thaps.), and **C** 3.3 μM Phorbol 12-myristate 13-acetate (PMA); and qRT-PCR was performed at 6 h (**A**) or 4 h (**B**, **C**) after treatment. Expression levels were normalized to GAPDH. *n* = 9 (**A**, **B**) and *n* = 7 (**C**) from ~30 pooled embryos per sample. Bars represent mean ± SEM. *p < 0.05; **p < 0.01; ***p < 0.001; ****p < 0.0001. Fold change (FC) is indicated.

To first determine if pro-apoptotic mRNAs were upregulated in zebrafish following diverse cellular stresses, we analyzed the relative expression of puma, noxa, bax, and bid (Fig. S1 depicts zebrafish orthology analysis) in 24 hours post fertilization (hpf) embryos exposed to either the genotoxic stress (30 Gy ionizing radiation, IR), ER stress (5 μM Thapsigargin, Thaps.), or oxidative stress (3.3 μM Phorbol 12-myristate 13-acetate, PMA). As with human cells [49, 50], puma has the strongest induction following IR, then noxa, followed by bax and bid have mild to no induction (Fig. 1A). Similar to IR, following Thaps. and PMA, both puma and noxa were significantly upregulated, however bax and bid were not induced (Fig. 1B, C). Together these data have indicated that puma and noxa are strongly transcriptionally regulated by cellular stresses. While the p53 family of stress mediators are largely controlled at the post-translational level, we analyzed the relative expression of p53, p63, and p73 after these stresses (Fig. S2). Only after IR did we observe increases in p53 mRNA, which is self-inducing (Fig. S3). p73 was significantly induced after IR (this induction is p53 dependent—Fig. S3), non-significantly induced with Thapsigargin treatment, and significantly reduced after PMA treatment. However, p63 mRNA was not significantly induced by any of the treatments.

### Generation of zebrafish null mutants

**Fig. 2:**
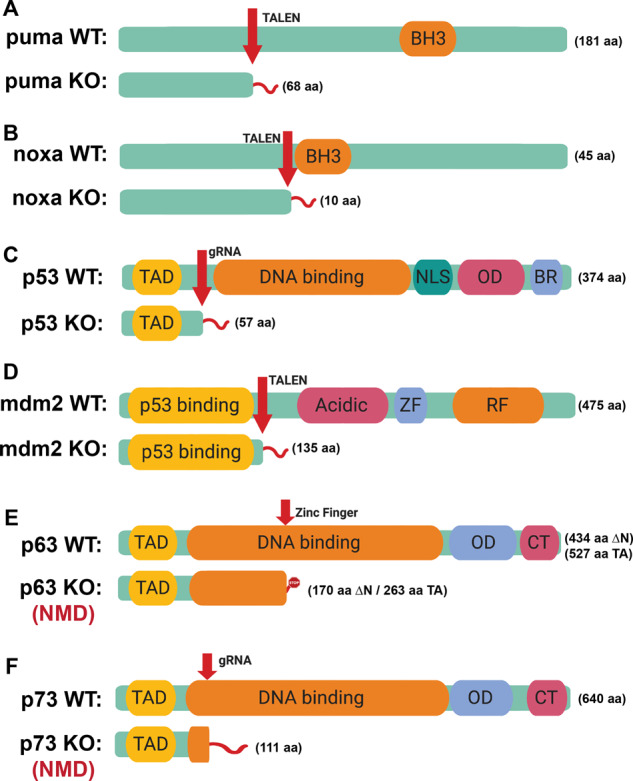
The protein structure of 6 mutant alleles in zebrafish generated by zinc finger, TALEN, or CRISPR-Cas9 gene editing. **A** Schematic of Puma wild-type and mutant proteins. (BH3, BH3 domain). **B** Schematic of Noxa wild-type and mutant proteins. **C** Schematic of p53 wild-type and mutant proteins. TAD transactivation domain, DNA-binding DNA-binding domain, NLS nuclear location signal, OD Oligomerization domain, BR basic region. **D** Schematic of Mdm2 wild-type and mutant proteins (p53 binding, p53-binding domain; Acidic, acidic domain; ZF, zinc finger domain; RF, ring finger domain). **E** Schematic of p63 wild-type and mutant proteins (CT, C-terminal region; p63 mutant transcripts undergo nonsense-mediated decay, NMD). **F** Schematic of p73 wild-type and mutants. Arrow points out the target site of zinc finger, TALEN, or CRISPR-Cas9 gene editing. Orange indicates the key domain in each protein. Light green bar labels full-length wild-type or in-frame truncated mutant protein and the length of amino acid (aa) sequence is indicated. Red tail indicates out-of-frame part of the truncated protein. The figure is created with BioRender.com.

To further pursue the importance of puma and noxa in the stress-induced apoptotic response in zebrafish, we generated zebrafish puma and noxa null alleles (Figs. 2, S4 and S5). PUMA and NOXA have been described to be transcriptionally induced in a p53 dependent as well as p63 and p73 dependent manner [19, 35–39, 51–53]. Therefore, to further evaluate the stress pathways we have also generated a p53 null allele (Figs. 2 and S6), as well as p63 and p73 null alleles (Figs. 2, S7 and S8) in zebrafish. To introduce an alternative mechanism of p53 induction and evaluate our new p53 null allele and puma null allele, we also generated a mdm2 null allele (Figs. 2 and S9). MDM2 is E3 Ubiquitin ligase. Mouse and zebrafish genetic experiments have established that deletion of the negative regulator of p53 [54, 55], MDM2, results in embryonic lethality due to unregulated activation of p53 and apoptosis. This lethality can be completely rescued in a p53 null background, solidifying the lethality is p53 dependent [56–58]. Important to this model is that p53 is hyper-activated in the absence of a true stress signal. Our overall strategy for all of these knockouts is to generate a small deletion or insertion 5′ in the coding region that results in a frame shift that truncates the protein (Fig. 2).

### Puma, but not noxa, is essential for p53-dependent induction of apoptosis following genotoxic stress

**Fig. 3:**
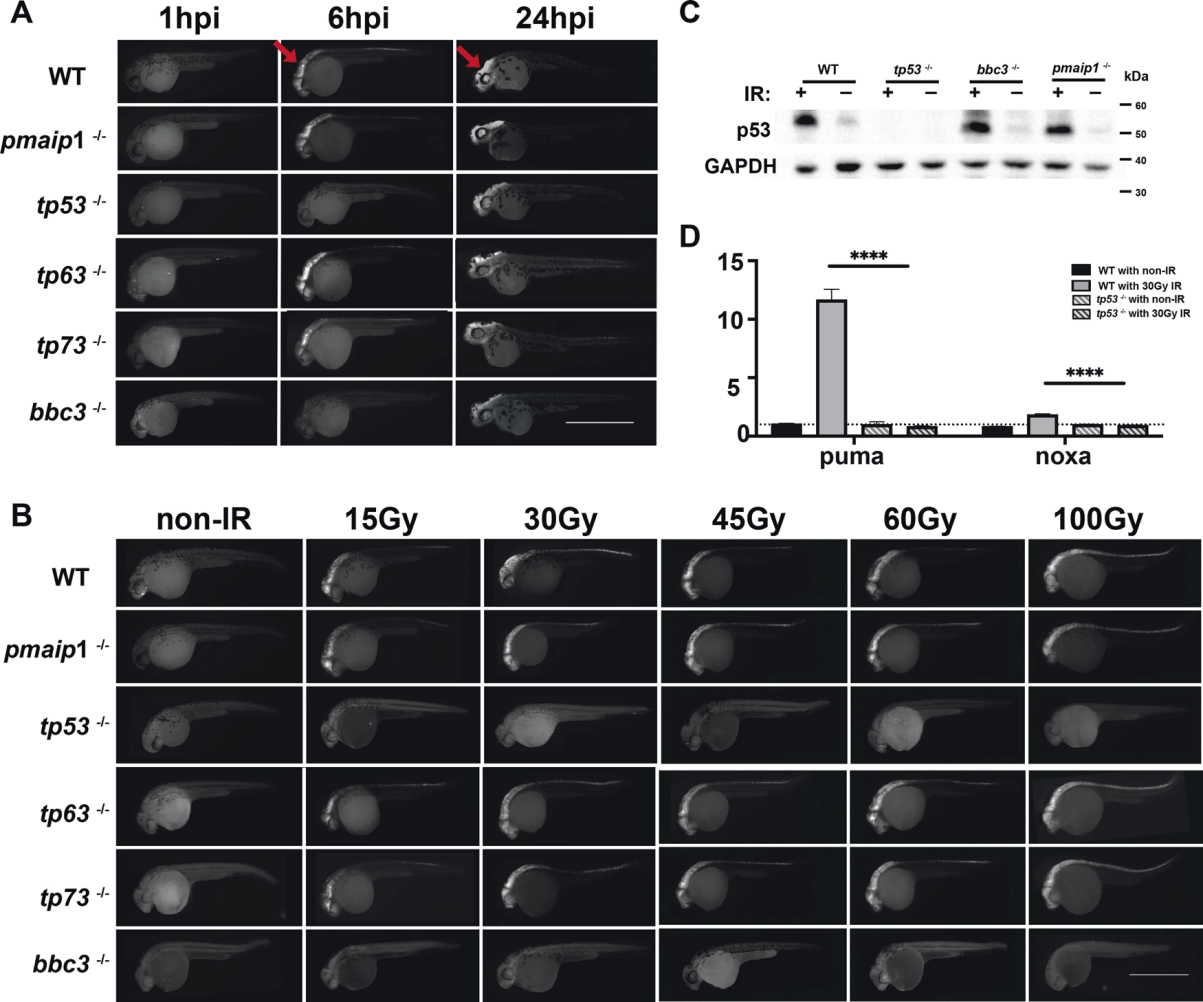
Loss of puma not noxa rescues IR-induced apoptosis in p53-dependent manner. **A** Anti-active Caspase-3 staining on 30 hpf (1 hpi and 6 hpi panel) or 48 hpf (24 hpi panel) embryos. 29 hpf wild-type, pmaip1^−/−^, tp53^−/−^, tp63^−/−^, tp73^−/−^ and bbc3^−/−^ zebrafish embryos were treated with 30 Gy IR-irradiation and fixed at 1 hour post treatment (1 hpi panel); 24 hpf embryos were treated with 30 Gy IR-irradiation and fixed at 6 h (6 hpi panel) or 24 h (24 hpi panel) after treatment. Arrows in WT points out active apoptotic area in head region in WT embryos for 6 hpi and 24 hpi. Scale bar, 1000 μM. **B** Anti-active Caspase-3 staining of 30 hpf (6 hpi) wild-type, pmaip1^−/−^, tp53^−/−^, tp63^−/−^, tp73^−/−^, and bbc3^−/−^ zebrafish embryos treated w/o IR (non-IR) and with 15, 30, 45, 60, and 100 Gy IR. Scale bar, 1000 μM. **C** p53 protein expression level after IR-induction. Western blot analysis was performed using protein extracts from 30 hpf (6 hpi) wild type, tp53^−/−^, bbc3^−/−^, and pmaip1^−/−^ zebrafish embryos with (30 Gy) or without IR treatment. **D** qRT-PCR analysis of puma and noxa after IR-irradiation in zebrafish embryos. 24 hpf wild-type or tp53^−/−^ zebrafish embryos were treated with 30 Gy IR-irradiation and RNA samples for qRT-PCR were harvested at 6 h after IR-irradiation. Expression levels were normalized to GAPDH. *n* = 6 from ~30 pooled embryos per sample. Bars represent mean ± SEM. ****p < 0.0001.

To investigate the genotoxic stress-induced apoptotic pathway in zebrafish, we have treated 24 hpf embryos with IR. Our data and others have indicated that IR-induced apoptosis primarily occurs in the neural tube of 24 hpf zebrafish embryos and is p53 dependent [43, 59–61]. This is consistent with mouse studies demonstrating apoptosis predominantly in the neural tube of 13.5 dpc embryos after IR treatment [62]. To determine if puma and noxa are required for the p53 dependent, as well as if p63 or p73 contribute to the apoptotic response in zebrafish, we treated zebrafish wild type, tp53^−/−^, tp63^−/−^, tp73^−/−^, bbc3^−/−^, and pmaip1^−/−^ embryos with 30 Gy IR-irradiation and stained for the apoptotic marker activated Caspase-3 at 1 hour post irradiation (hpi), 6 hpi, and 24 hpi. In wild-type embryos, we do not observe apoptosis within 1 h, but do by 6 hpi and this persists into 24 hpi (Figs. 3A and S10). We also performed TUNEL staining to validate the active Caspase-3 apoptotic staining on untreated and IR treated wild-type embryos (Fig. S11). For p53 null, we do not observe apoptosis at 1 hpi or 6 hpi, but do observe apoptosis at 24 hpi (Figs. 3A and S10). This indicates the primary apoptotic response at 6 hpi is in a p53 dependent manner; however, the later 24 hpi apoptosis is p53 independent. Interestingly loss of puma, but not noxa, resulted in the loss of apoptosis at 1 hpi and 6 hpi, but not 24 hpf similar to p53 loss (Figs. 3A, S10). These data suggest that puma alone, but not noxa, is an essential mediator of IR-induced p53-dependent apoptotic response. Note, puma loss does not alter the 24 hpi apoptosis suggesting this apoptosis is through a p53/puma independent mechanism. p63 or p73 null embryos undergo apoptosis similar to wild-type and noxa null (Figs. 3A and S10) embryos, suggesting they are not essential for IR-induced apoptosis. The fact that puma alone mediates the IR-induced apoptotic response was surprising and raised the possibility that puma loss abrogates apoptosis at milder doses of IR, but at stronger doses noxa may also contribute. Therefore, we treated wild type, tp53^−/−^, tp63^−/−^, tp73^−/−^, bbc3^−/−^, and pmaip1^−/−^ embryos with either 0, 15, 30, 45, 60, and 100 Gy of IR and stained them with the active Caspase-3 and apoptotic dye Acridine orange (AO) (Figs. 3B and S12). The puma null was equally effective at preventing IR-induced apoptosis as the p53 null at all doses, again suggesting that puma is the essential mediator of p53-dependent apoptosis. To further evaluate the lack of involvement of noxa in IR-induced apoptosis, we compared wild type and pmaip1^−/−^ embryos with a 15 Gy low dose and observed a very mild apoptosis in the wild type embryos and similar level of apoptosis in pmaip1^−/−^ embryos (Figs. 3B and S12); suggesting noxa is not essential for IR-induced p53-dependent apoptosis in 24 hpf zebrafish embryos. Following IR treatment, the p53 protein levels accumulate due to the inhibition of interaction with the E3 ubiquitin ligase mdm2, thereby extending the half-life of the p53 protein. To address the possibility that puma loss influences p53 protein accumulation, we performed a western blot for p53 following IR treatment. p53 protein accumulated to equivalent levels in the wild type, bbc3^−/−^ and pmaip1^−/−^, but not p53^−/−^ following IR treatment (Fig. 3C). This indicates that the loss of puma has no influence on p53 protein levels. Further, using qRT-PCR of RNA extracted from p53 wild-type and p53 null embryos either untreated or 6 h after 30 Gy IR we demonstrated that puma and noxa mRNA induction is in a p53-dependent manner (Fig. 3D).

### Puma, but not noxa, is essential for mdm2-null induced p53-dependent apoptosis

**Fig. 4:**
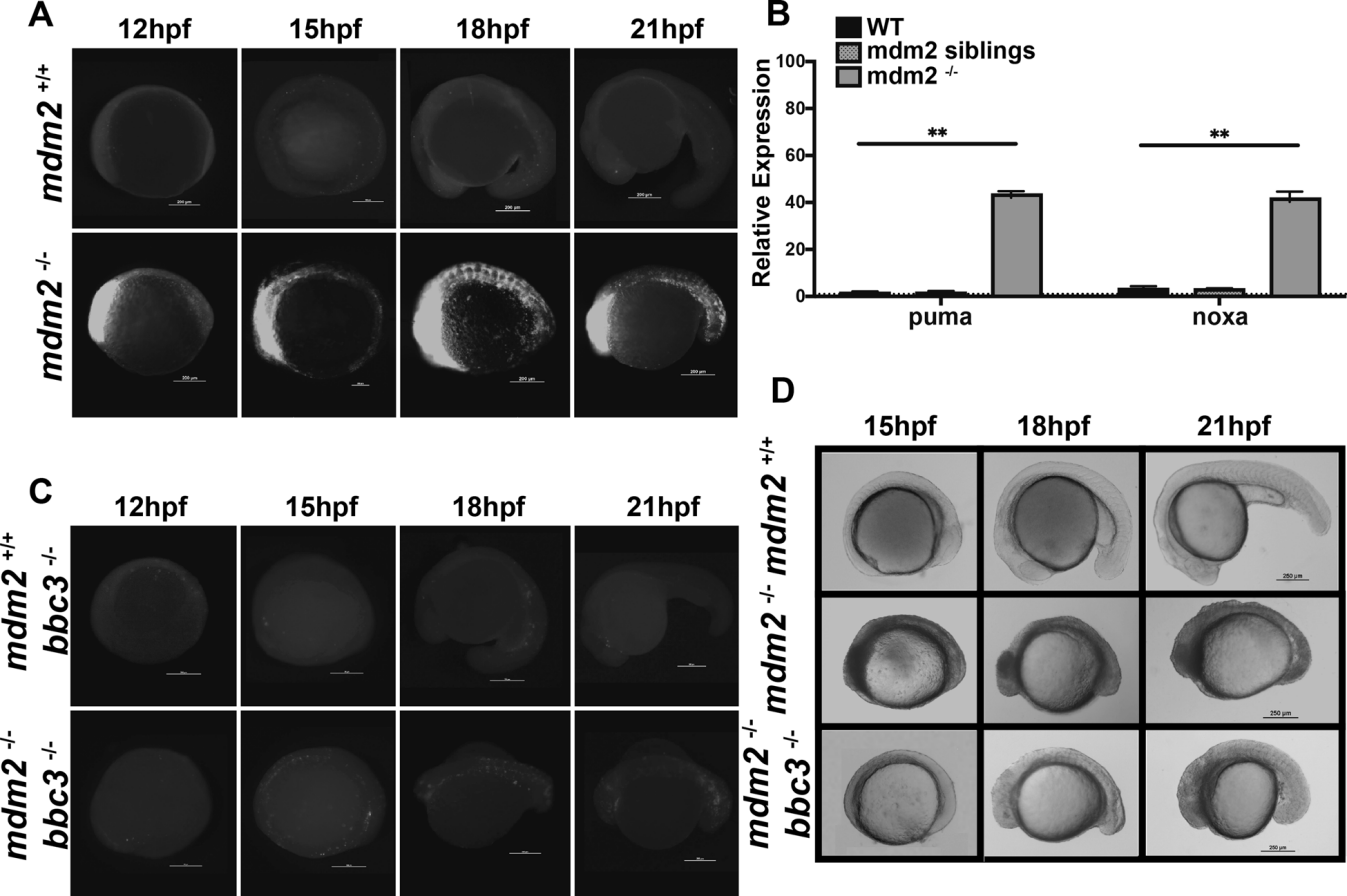
Loss of puma rescues mdm2-null induced apoptosis at early embryonic stage. **A** Anti-active Caspase-3 staining on 12, 15, 18, and 21 hpf mdm2^+/+^ and mdm2^−/−^ zebrafish embryos. **B** qRT-PCR analysis of puma and noxa in 15 hpf mdm2^+/+^, mdm2 siblings (mdm2+/+ and mdm2^+/−^) and mdm2^−/−^ zebrafish embryos. *n* = 3 from ~30 pooled embryos per sample. Bars represent mean ± SEM. **p < 0.01. **C** Anti-active Caspase-3 staining on 12, 15, 18, and 21 hpf mdm2^+/+^ bbc3^−/−^ and mdm2^−/−^ bbc3^−/−^ zebrafish embryos. **D** Gross images of 15, 18, and 21 hpf mdm2^+/+^, mdm2^−/−^, and mdm2^−/−^ bbc3^−/−^ zebrafish embryos.

There are a number of stresses, beyond genotoxic stress, that can activate a p53-dependent apoptosis. The role of Puma in these stresses is unclear and potentially mediated by other apoptotic regulators. To investigate if puma is the sole mediator of p53-induced apoptosis, we will employ the mdm2 null zebrafish. Within this model, mdm2 loss circumvents the need for signaling pathways involved in cellular stresses and induces a universal p53 activation response. Consistent with mouse, loss of mdm2 in zebrafish results in an early embryonic lethality morphologically identifiable prior to 15 hpf due to extensive apoptosis as early as 12hpf (Fig. S9E and 4A). Further, this lethality is completely rescued by loss of p53 (Fig. S9F, G). By qRT-PCR we determine that both puma and noxa are strongly induced in mdm2 null embryos (Fig. 4B), suggesting they are likely involved in the p53-dependent apoptotic response. To determine if puma and/or noxa are essential mediators of the p53-dependent apoptotic response, we generated double mutants, mdm2^−/−^; bbc3^−/−^ and mdm2^−/−^; pmaip1^−/−^ embryos and accessed their apoptotic and morphological phenotypes. Loss of noxa had no effect on the apoptosis or the morphological phenotype (data not shown). Loss of puma completely abrogated the apoptotic response and mildly rescued the phenotype (Fig. 4C, D). The mild rescue of the phenotype suggests that other p53-induced biological outcomes (e.g., cell cycle arrest) are also influential in the phenotype. Importantly this indicates that puma, but not noxa, is the essential mediator of the p53-dependent apoptotic response.

### ER stress-induced apoptosis response requires the involvement of p63 and puma but not noxa, p53 and p73

**Fig. 5:**
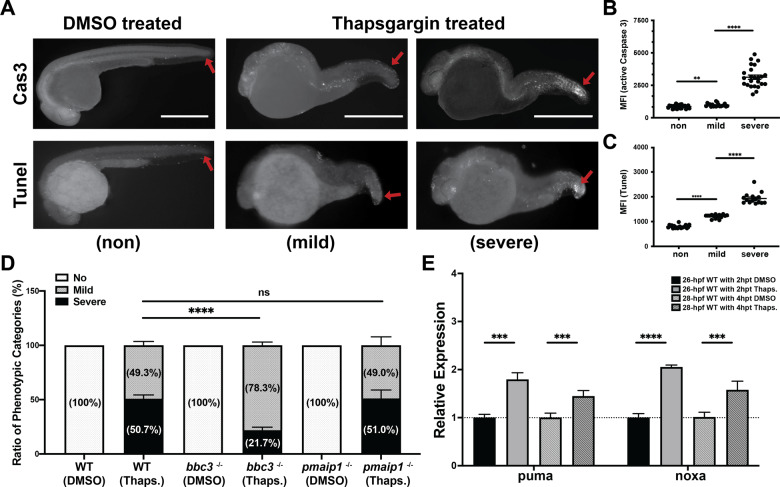
Loss of puma partially rescues Thapsigargin (Thaps.)-induced apoptosis at early embryonic stage. **A** Anti-active Caspase-3 (The Upper Panel) and TUNEL (The Lower Panel) staining on 28 hpf (4 h post treatment) wild-type zebrafish embryos with DMSO alone or with DMSO plus 5 μM Thaps. Representative figures showing phenotypic categories of the apoptotic severity. Arrows points out apoptotic area in tail region in WT embryos with DMSO alone or with DMSO plus 5 μM Thaps for 4 h. Scale bar, 1000 μM. **B** Quantification of the fluorescence intensity of tail region of DMSO-treated and Thaps. treated embryos in mild and severe categories with anti-active Caspase-3 staining. Each dot represents mean fluorescence intensity (MFI) of the tail region of individual embryos from three independent experiments. Bars represent mean ± SEM. **p < 0.01.****p < 0.0001. **C** Quantification of the fluorescence intensity of tail region of DMSO-treated and Thaps. treated embryos in mild and severe categories for TUNEL staining. Each dot represents mean fluorescence intensity (MFI) of the tail region of individual embryos from two independent experiments. Bars represent mean ± SEM. ****p < 0.0001. **D** Loss of puma (not noxa) partially rescued Thaps. induced apoptosis at 24 hpf. Ratio of phenotypic categories in wild type, bbc3^−/−^ and pmaip1^−/−^ zebrafish embryos. *n* = 7 (wild type and bbc3^−/−^) and *n* = 4 (pmaip1^−/−^) from pooled embryos per sample. The total number of Thaps. treated embryos: wild type > 1000, bbc3^−/−^ > 900 and pmaip1^−/−^ > 550. Bars represent mean ± SEM. ****p < 0.0001. **E** qRT-PCR analysis of puma and noxa after DMSO or 5 μM Thapsigargin treatment in 24 hpf zebrafish embryos across time (2hpt and 4hpt). Expression levels were normalized to GAPDH. *n* = 5 (26-hpf WT with or w/o 2 h post Thaps. treatment) and *n* = 9 (28-hpf WT with or w/o 4 h post Thaps. treatment) from ~30 pooled embryos per sample. Bars represent mean ± SEM. ***p < 0.001; ****p < 0.0001.

ER stress has been shown to be important in a number of disease states including but not limited to retinal degeneration, diabetes, obesity, and neurological disorders [2]. As with IR, ER stress has multiple outputs, one of them being apoptosis. Thapsigargin (Thaps.) is a well-studied ER stressor [63, 64]. Previous studies in zebrafish indicated that ER stress, through IRE-1 and Perk but not Chop, activates p63 and Puma to induce an apoptotic response primarily in the epithelium [19]. However, the validity of the Puma and p63 involvement are controversial due to the use of morpholinos that have potential off-target effects. Consistent with published data, we observe similar morphology changes in Thapsigargin-treated embryos and elevated apoptosis by active Caspase-3 staining and TUNEL staining in the epithelial layer, particularly in the growing tail tip (Fig. 5A). While all treated embryos have a morphological curved body phenotype, we did observe that about half have a mild apoptosis in the tail tip region (49.3%; lower mean florescent intensity Fig. 5A–D) and half have a severe apoptosis (50.7%; higher mean florescent intensity; Fig. 5A–D). By qRT-PCR, we observed that puma, as well as noxa, are transcriptionally induced following treatment with Thaps. both at 2 and 4 hours post treatment (hpt) (Fig. 5E); suggesting both of them are mediators of the apoptotic response. To define if puma and noxa are required for the ER stress-induced apoptotic response, we performed the Thaps. treatment on bbc3^−/−^ and pmaip1^−/−^ embryos. While the loss of noxa had no effect on apoptosis following Thaps. treatment (Fig. 5C), puma loss significantly reduced apoptosis from 50.7 to 21.7%. It suggests that puma, but not noxa, is important in the ER stress-induced apoptotic response at 24 hpf embryos. It also suggests that other factors are involved. Loss of either noxa or puma did not impact the overt morphological change (mild phenotype) which is likely due to ER stress-induced non-apoptotic outcomes.

To further confirm these findings, we treated wild-type and bbc3^−/−^ embryos with Brefeldin A (BFA), an additional ER stress inducing compound [19]. Similar to Thapsigargin, BFA induced a similar morphological phenotype and a mild and severe apoptotic response (Fig. S13A, B) with a longer treatment time (6 hpt). It also induced puma and noxa transcriptionally at 6 hpt (Fig. S13C) and the apoptosis was suppressed in a bbc3^−/−^ background (88.3–2.3%, Fig. S13D). The almost complete suppression of apoptosis could suggest that the ER stress response to Thaps. and BFA are slightly different, with BFA being more puma dependent. Together these data, supports that ER stress induces a puma-dependent apoptotic response.

**Fig. 6:**
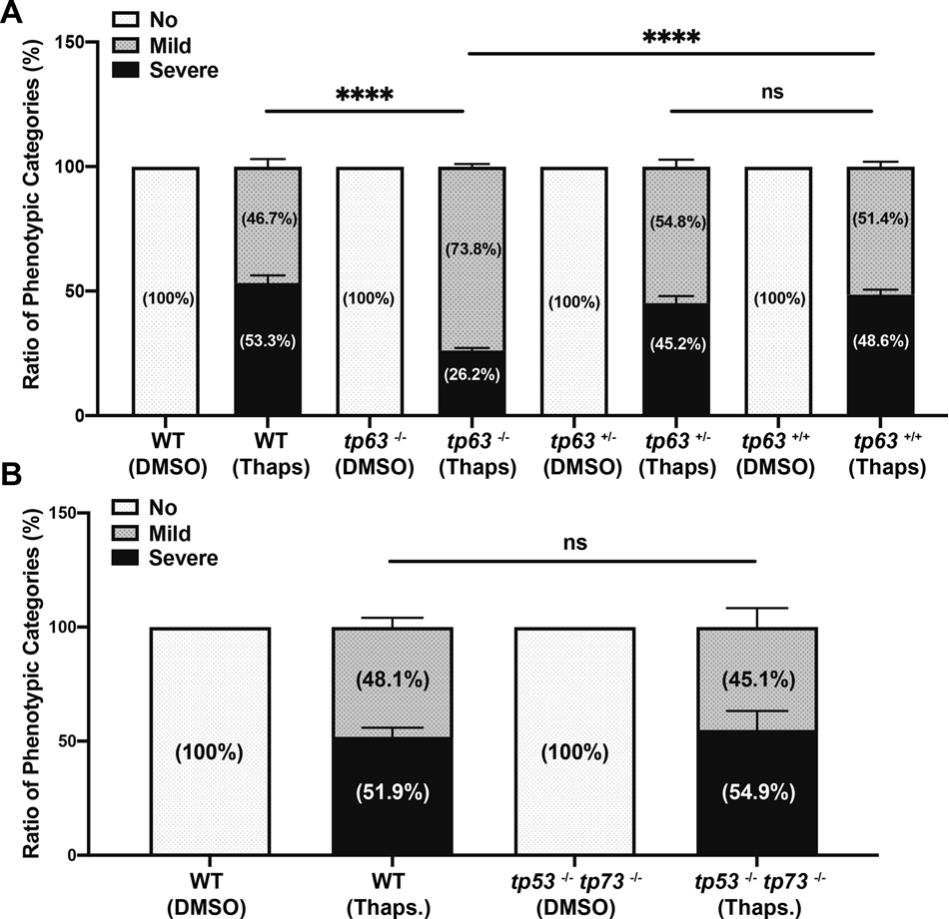
Loss of p63 (not p53 and p73) partially rescued Thaps. induced apoptosis at early embryonic stage. **A** Loss of p63 partially rescued Thaps. induced apoptosis at 24 hpf. Ratio of phenotypic categories of the apoptotic severity in wild type, tp63^−/−^, sibling tp63^+/−^ and sibling tp63^+/+^ zebrafish embryos at 4 h after DMSO or Thaps. treatment. Embryos from the intercross of heterozygous-mutant tp63 adults were genotyped after sorted based on the apoptotic severity at tail region. **B** Loss of p53 and p73 cannot rescue Thaps. induced apoptosis at 24 hpf. Ratio of phenotypic categories in 24 hpf wild-type and tp53^−/−^ tp73^−/−^ zebrafish embryos at 4h after DMSO or Thaps. treatment. *n* = 6 (**A**) and *n* = 4 (**B**) from pooled embryos per sample. The total number of Thaps treated embryos: wild type > 660, tp63^−/−^ > 360, tp63^+/−^ > 700, tp63^+/+^ > 320 and tp53^−/−^ tp73^−/−^ > 420. Bars represent mean ± SEM. ****p < 0.0001.

To determine if p63 is involved in the ER stress response, we treated tp63^−/−^ embryos with Thaps. While p63 null embryos display morphological phenotypes at 3.5 dpf, we did not observe any difference in morphology or apoptosis in 24 hpf p63 homozygous null embryos vs. wild-type embryos (Fig. S7F, G). However, we did observe a significant reduction in apoptosis in p63 null embryos following Thaps. treatment (Fig. 6A) when compared with wild type and sibling tp63+/+ (internal control). This reduction was similar to that observed in the puma null embryos (Fig. 5D). To further test whether p53 and p73 are involved in the ER-induced apoptosis response, we treated double null embryos (tp53^−/−^; tp73^−/−^) with Thaps. and observed no significant change in apoptosis (51.9–54.9%, Fig. 6B). Together these data indicate that the ER-induced apoptotic response is partially through p63/puma axis, but is in a p53, p73, or noxa-independent manner in 24 hpf zebrafish embryos.

### Oxidative stress-induced p63 mediated puma/noxa-dependent apoptosis response that does not require the involvement of p53 and p73

**Fig. 7:**
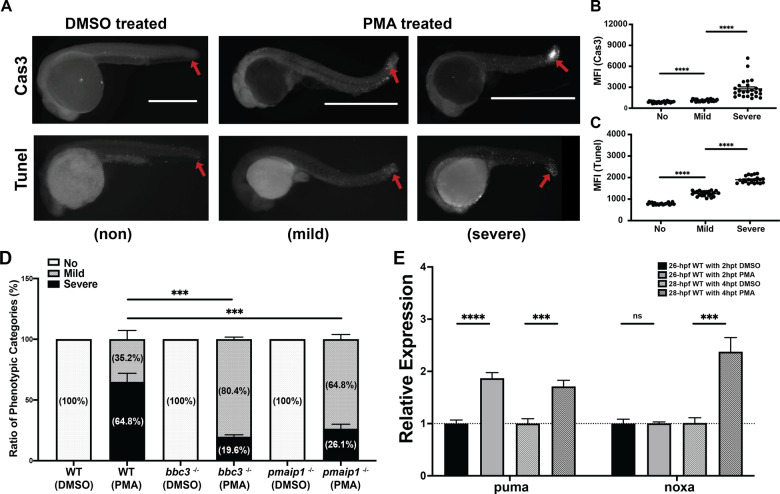
puma and noxa are required for PMA-induced apoptosis. **A** Anti-active Caspase-3 (The upper panel) and TUNEL (The Lower Panel) staining on 28 hpf (4 h post treatment) wild-type zebrafish embryos with DMSO or DMSO plus 3.3 μM PMA. Representative figures showing phenotypic categories of the apoptotic degree. Arrows points out apoptotic area in tail region in WT embryos with DMSO alone or with DMSO plus 3.3 μM PMA for 4 h. Scale bar, 1000 μM. **B** Quantification of fluorescence intensity of tail region of DMSO-treated and PMA-treated embryos in mild and severe categories with anti-active Caspase-3. Each dot represents MFI of the tail region of individual embryos from three independent experiments. Bars represent mean ± SEM. ****p < 0.0001. **C** Quantification of the fluorescence intensity of tail region of DMSO-treated and PMA-treated embryos in mild and severe categories for TUNEL staining. Each dot represents mean fluorescence intensity (MFI) of the tail region of individual embryos from two independent experiments. Bars represent mean ± SEM. ****p < 0.0001. **D** Loss of both puma and noxa partially rescued PMA-induced apoptosis at 24 hpf. Percentage of phenotypic categories in wild type, bbc3^−/−^ and pmaip1^−/−^ zebrafish embryos. *n* = 4 (wild type and bbc3^−/−^) and *n* = 7 (pmaip1^−/−^) from pooled embryos per sample. The total number of PMA-treated embryos: wild type > 600, bbc3^−/−^ > 440, and pmaip1^−/−^ > 800. Bars represent mean ± SEM. ***p < 0.001. **E** qRT-PCR analysis of puma and noxa after DMSO or 3.3 μM PMA treatment in 24 hpf zebrafish embryos across time (2hpt and 4 hpt). Expression levels were normalized to GAPDH. *n* = 5 (26 hpf WT) and *n* = 7 (28 hpf WT) from around 30 pooled embryos per sample. Bars represent mean ± SEM. ***p < 0.001; ****p < 0.0001.

**Fig. 8:**
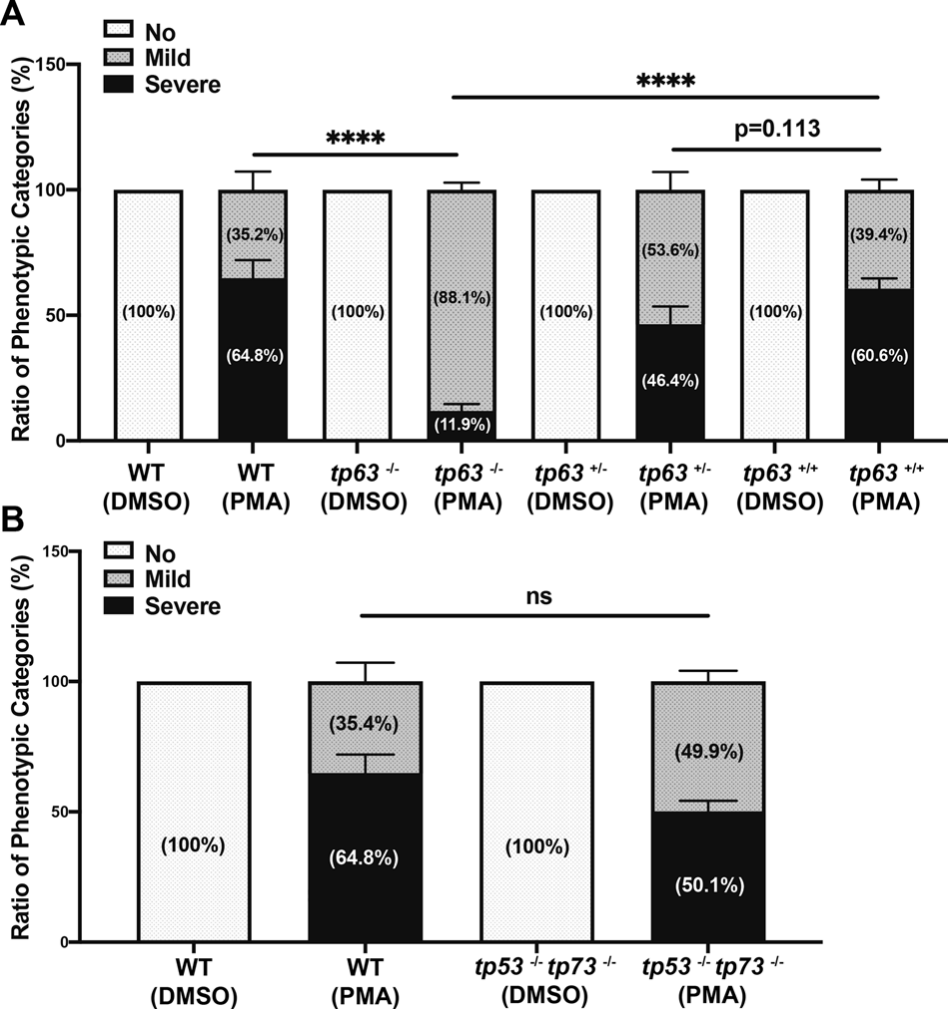
*p63*, but not *p53* and *p73*, are required for PMA-induced apoptosis. **A** Percentage of phenotypic apoptotic categories in wild type, *tp63*^−/−^, sibling *tp63*^+/−^, and sibling *tp63*^+/+^ zebrafish embryos at 4 h after treatment with DMSO or PMA. **B** Percentage of phenotypic categories in 24 hpf wild type and *tp53*^−/−^ *tp73*^−/−^ zebrafish embryos at 4 h after DMSO or PMA treatment. *n* = 6 (**A**) and *n* = 5 (**B**) from pooled embryos per sample. The total number of Thaps treated embryos: wild type > 600, *tp63*^−/−^ > 140, *tp63*^+/−^ > 390, *tp63*^+/+^ > 180, and *tp53*^−/−^ *tp73*^−/−^ > 370. Bars represent mean ± SEM. ****p < 0.0001.

To decipher the oxidative stress-induced apoptotic pathway, we treated zebrafish embryos with PKC activator PMA. We observed a morphological change and elevated active Caspase-3 and TUNEL staining following PMA treatment of 24 hpf embryos (Fig. 7A). Like ER stress, the apoptosis is predominant in the embryonic epithelium, and we observe a mild and severe apoptotic phenotype at the tail tip region (Fig. 7A–C). To investigate if puma or noxa are required for the oxidative stress-induced apoptosis, we treated bbc3^−/−^ and pmaip1^−/−^ embryos with PMA and stained for active Caspase-3. Interestingly, both puma and noxa are important for the PMA-induced apoptotic response (64% vs 19.6% or 26.1%, respectively, Fig. 7D), suggesting that both puma and noxa are important in oxidative stress-induced apoptosis response for 24 hpf zebrafish embryos. While we observed both puma and noxa are transcriptionally induced following 4-h PMA treatment, puma is induced earlier and can be observed following 2-h PMA treatment (Fig. 7E). However, loss of either puma or noxa did not rescue some non-apoptotic morphological outcomes induced by PMA treatment. In addition, we observed a significant reduction in apoptosis in tp63 null embryos treated with PMA treatment from 64.8 to 11.9% (Fig. 8A) that is better than the rescue of loss of puma or noxa. We did not observe a significant reduction in apoptosis in p53/p73 double null embryos after 4-h PMA treatment (Fig. 8B). These data suggest that PMA-induced apoptosis is p63, but not p53 or p73, dependent; however, unlike ER stress, the apoptotic response requires the involvement of both puma and noxa.

## Discussion

### Genotoxic stress pathway

Consistent with previous reports, we have demonstrated that genotoxic stress induces a robust p53-dependent apoptosis in the neural tube of 24 hpf zebrafish embryos [60, 61]. This is consistent with mouse data demonstrating predominantly neural tube apoptosis in 13.5 dpc embryos after irradiation [62]. This suggests either that the neural tissues are poised for apoptosis or that there is a unique p53 transcriptional profile in the neural tissues that drives apoptosis preferentially. This could explain why so many DNA repair deficient or genomic instability associated diseases have neural associated defects, such as ataxia [65–68]. Our data indicate that Puma is the key mediator of p53-dependent apoptosis due to genotoxic stress. Puma inhibitors could potentially be used to provide a neuro-protectant effect. Interestingly, we observed that there is a first wave of apoptosis that is p53/puma dependent, and a later wave 24 hpi that is p53/puma independent. The molecular mechanism of this second wave is still unknown but could be a consequence of cells undergoing mitotic slippage while still containing broken chromosomes. The fact that Caspase-3 is activated indicates it is a programed pathway which should be explored. The rescue of the mdm2 null induced apoptosis, indicates Puma is the key mediator of p53-induced apoptosis. This brings into question of why is Noxa evolutionarily conserved and induced following genotoxic stress, and why does it alone not induce apoptosis when induced? While mammalian studies often refer to Puma and Noxa being essential for p53-dependent apoptosis, the data in mouse studies also strongly suggest that Puma is the key regulator of p53-dependent apoptosis [49, 50, 69]. While we have focused on elevation of the noxa mRNA transcript, the post-translational modifications of NOXA protein have been shown to influence its apoptotic function [70]. Potentially under genotoxic stress noxa protein is not activated post-transcriptionally.

### The response to ER stress

Many pathological studies have recently demonstrated that ER stress is central to many diseases. We have demonstrated that the full ER stress apoptotic response in the epidermis requires activation of p63, but not p53 or p73. This is consistent with zebrafish morpholino data indicating the ER stress utilizes p63 for apoptosis in the epidermis [19]. Our data also indicate that puma, but not noxa, is required for ER stress-induced apoptosis in the epidermis. The epidermal apoptosis is likely because p63 has been described as important for maintenance of the epithelium and is predominantly expressed there (Fig. S7E) allowing for puma induction preferentially in the epithelium. The loss of p63 and puma did not completely abrogate the apoptotic response suggesting additional stress-induced apoptotic pathways, not involving the p53 family or puma/noxa. Genetic studies using mutants in other BH3 only proteins may help identify these other pathways. Interestingly, the ER stress response to BFA is almost completely mediated by p63/Puma, suggesting differences in drug induce ER stress responses. Future studies addressing the importance of IRF, ATF6, and PERK upstream of p63, would be useful to delineate this pathway as well as tissue-specific differences in ER stress responses.

### Reactive oxygen stress response

While reactive oxygen stress is often associated with genotoxic stress, it is unique. Towards this, we demonstrate that oxidative stress-induced apoptosis is mediated by p63 and not p73 or the genotoxic stress mediator p53. Unlike ER stress, oxidative stress in the epithelium does involves both puma and noxa. What is unique about oxidative stress to require both factors are unknown? It is worth noting that the actual outcomes of PMA treatment are quite prolific in response; for example, PMA does activate NF-κB in some cell types [71, 72]. This could confound if the pathway is purely oxidative stress involved. This does not take away for the unique apoptotic aspects in that noxa is induced in all stresses tested, but only with PMA is noxa required for the full apoptotic response.

### Therapeutic application

While p53 has been most well studied in the context of cancer, p53 is also involved in many developmental and non-cancerous diseases. The potential for inhibition of p53 has been contemplated, however not pursued due to concerns over the impact on cancer predisposition. For example, Treacher Collins Syndrome (TCS) is a genetic disease associated with ribosomal stress-inducing p53-dependent apoptosis predominantly in the neural crest cell resulting in craniofacial defects. Interestingly the TCS mouse phenotype is abrogated in a p53 null background. While inhibition of p53 may have long-term cancer implications, loss of Puma both in mouse and zebrafish do not form tumors and therefore inhibition of puma could be a very effective therapeutic to prevent stress-induced apoptosis-associated diseases.

### Zebrafish to understand cellular stress associated diseases

While mouse is the dominant model in most disease research, zebrafish provides numerous advantages that have propagated its applications in biomedical research. The major limitation is genetic reagents since this is a relatively young model system. Within this study, we provide six new zebrafish null alleles that can be used in the biomedical community. Toward monitoring the consequence of stress signals the transparency of zebrafish embryos and adults allows for single-cell analysis of fluorescent reporter lines in real time in a live animal [73, 74]. Towards this, recently a zebrafish ER stress reporter line has been generated that allows for in vivo visualization of ER stress [75]. In conjunction with disease models this could assist in understanding the pathology of the disease. In addition, zebrafish are highly amenable to chemical treatments as well as drug screens for suppressor of stress-induced phenotypes.

## Materials and methods

### Zebrafish lines and maintenance

All zebrafish work was performed in the Zebrafish Research Facility (ZRF) of the University of Alabama at Birmingham (UAB). Adult fish and embryos are maintained as described by Westerfield M (1995) [76] by the ZRF Animal Resources Program which maintains full AAALAC accreditation and is assured with OLAW. All knockout lines are generated on the AB stain. All animal studies have UAB IACUC approval.

### Transcript IDs in homology analysis

For the homology analysis, we used the following transcripts: hPUMA (ENST00000439096.3), hNOXA (ENST00000316660.7), hBMF (ENST00000397573.5), hBid (ENST00000622694.5), hBim (ENST00000308659.12), hHrk (ENST00000257572.5), hBik (ENST00000216115.3), hBad (ENST00000309032.8), zPuma (ENSDART00000137918.3), zNoxa (ENSDART00000123131.3), zBmf (ENSDART00000060713.5), zBid (ENSDART00000100716.7), zBim (ENSDART00000114318.3), zHrk (ENSDART00000132567.2), zBad (Bada: ENSDART00000125349.3 and Badb: ENSDART00000077219.5), tp53 (ENSDART00000051549.5), tp63 (TA: ENSDART00000163541.2 and ΔN: ENSDART00000065135.5), and tp73 (ENSDART00000124737.3).

### Generation of new knockout alleles

Gene knockouts were generated as described previously [77]. Zinc Finger, TALEN, or gRNA target sites were identified using the ZiFiT Targeter software developed by the Zinc Finger Consortium (http://zifit.partners.org/ZiFiT/), TAL Effector Nucleotide Targeter 2.0 (https://tale-nt.cac.cornell.edu/), and the Zhang lab gRNA design tool (http://crispr.mit.edu); respectively (target sites listed in figures). OPEN Pool ZFN were assembled into the pH3U3-mcs plasmid and selected using the omega knockout bacterial hybrid selection strain USO hisB-pyrF-rpoZ (Addgene #18049) [78]. TALENs were assembled using The Golden Gate TALEN and TAL effector kit (Addgene #1000000016) [79]. The CRISPR gRNA sequences were clones into pDR274 (Addgene 42250). The cas9 mRNA was transcribed from pT3TS-nCas9n (Addgene 46757) [80]. After cloning specific target plasmids/guides into pCS2 variant vector, mRNA was generated by in vitro transcription off NotI linearized DNA using the Invitrogen mMESSAGE mMACHINE™ SP6 Transcription Kit (Fisher Scientific AM1340) and purified with the MEGAclearTM Transcription Clean Up Kit (Fisher Scientific AM1908). Approximately 1–2 nl of nuclease mRNA (or sgRNA/Cas9 mRNA) were microinjected into the yolk of one-cell-stage zebrafish embryos. For indel efficiency evaluation, genomic DNA was extracted from ~24 3 dpf-injected embryos and evaluated with HRM (see below). The remaining embryos (F0s) from the clutches were raised. Out of frame indels identified in F1 progeny were maintained and propagated. To “cleanup” genetic background all lines were breed at least two generation to the wild-type strain AB.

### Identification of mutated alleles, nonsense-mediated decay, and alternative splicing

To determine if the mutated allele is undergoing nonsense-mediated decay or alternative splicing, a small piece of tail was cut from a single heterozygous fish (of each allele). RNA was extracted from each tail using Trizol Reagent (Life Technologies, 15596026), and cDNA was synthesized from each RNA sample using the High-Capacity cDNA Reverse Transcription Kit (Life Technologies, 4368814). The cDNA was PCR amplified using Takara Ex Taq DNA Polymerase (Takara Bio, RR001A), purified with the Promega Wizard SV Gel and PCR Cleanup System (Promega, A9282), and examined on a 1% agarose gel (for examining alternative splicing) and sequenced by the UAB Heflin Center for Genomic Sciences Sanger Sequencing Core. The mutated allele was determined to have undergone complete nonsense-mediated decay if only the wild-type sequence was detected in the sequence chromatogram.

### Genotyping with high-resolution melt analysis (HRMs)

To isolate genomic DNA from adults, tail clippings from each fish were incubated at 98 °C for 20 min in 40 µl 25 mM NaOH in a 96-well plate; then neutralized with 40 µl of 40 mM Tris-HCl. Early-stage or stained embryos were incubated at 55 °C 2 h in 25 µl ELB (10 mM Tris pH 8.3, 50 mM KCl, 0.3% Tween 20, 0.3% NP40, 1 mg/ml Proteinase K) in 96-well plates; then incubated at 95 °C for 15 min to inactivate the Proteinase K. PCR reactions contained 1 µl of LC Green Plus Melting Dye (Biofire Defense, BCHM-ASY-0005), 1 µl of 10x enzyme buffer, 0.2 µl of dNTP Mixture (10 mM each), 0.3 µl of MgCl2, 0.3 µl of each primer (10 µM), 1 µl of genomic DNA, 0.05 µl of Genscript Taq (E00101), and water up to 10 µl. The PCR reaction protocol was 98 °C for 30 s, then 45 cycles of 98 °C for 10 s, 59 °C for 20 s, and 72 °C for 15 s, followed by 95 °C for 30 s and then rapid cooling to 4 °C. Following PCR, melting curves were generated and analyzed using the LightScanner instrument (Idaho Technology) over a 65–95 °C range. Primers used for identifying zebrafish knockout lines are listed in Supplementary Table 1.

### IR-irradiation and apoptotic detection

Embryos were placed at the closest position to the source of IR in a X-RAD 320 X-ray irradiator to expose the embryos to ~4.2 Gy/min. Apoptosis was assayed following IR treatment by AO or active Caspase-3 staining. For AO staining [74], embryos were incubated in 50 mg/ml of AO (Sigma) for 45 min followed by five consecutive 5-min washes. Images were immediately taken using dissecting microscopy with 488 nm wavelength filter.

### Drug treatment

Overall, 1.5 µl Thapsigargin (10 mM stock in DMSO, Sigma), 3 µl BFA (10 mM stock in DMSO, Sigma), and 1 µl PMA (10 mM stock in DMSO, Sigma) were added into 3 mL of sterile E3 egg water to prepare working solutions with a final concentration of 5 µM Thapsigargin [19], 10 µM BFA or 3.3 µM PMA. Approximately 30 24 hpf embryos (±15 min) were placed in each well of six-well tissue culture plates (BD Falcon). For immediate apoptosis induction, embryos were left for 4 h (Thapsigargin and PMA) or 6 h (BFA) in the dark at 28.5 °C, processed for active Caspase-3 whole-embryo staining and sorted based on phenotypic categories (described in figures) to define the degree of severity.

### Whole-embryo immunohistochemistry staining

Embryos were fixed in 4% paraformaldehyde overnight at 4 °C and then permeabilized for at least 2 h in methanol (for anti-active Caspase-3 staining) or for 7 min in acetone (for anti-p63 staining). After 1 h blocking, embryos were incubated in primary antibody overnight at 4 °C. Anti-active Caspase-3 antibody (BD, 559565) was used at a dilution of 1:500 and anti-p63 primary antibody (Abcam, ab735) at 1:200. For Caspase-3 detection, the Alexa 488 goat anti-rabbit secondary antibody was used and for p63 detection, an Alexa 488 Donkey anti-mouse secondary antibody was used at a dilution of 1:200 for 2 h at room temperature or overnight at 4 °C. Subsequently, embryos were washed and stained in DAPI for 10 min (if nucleus measurement necessary) prior to imaging.

### Light, immunofluorescence, and confocal imaging

Embryos were dechorionated at described stages with incubation in 0.03% pronase (Sigma P5147) for 6 min and anesthetized using 0.4% tricaine. In a 60 × 15 mm Falcon petri dish (light and immunofluorescence imaging) and glass-coverslip-bottomed dish (confocal imaging), embryos are mounted in 1% low melting agarose. Gross images and images with AO or anti-active Caspase-3 staining were taken on a SMZ-18 Zoom Stereo Microscope. For quantification, all images were acquired at the same magnification, laser power, exposure time, and gain. Images with anti-p63 staining were taken on a Nikon A1 inverted confocal microscope and ~100-μm Z-stacks at 3.5-μm intervals were obtained. After each embryo was imaged, embryos were removed from the agarose to generate genomic DNA for genotyping. Further figure processing and analysis was performed using Nikon NIS Element and ImageJ.

### Quantitative real-time PCR

RNA was isolated from at least 30 pooled whole embryos using the Qiagen RNeasy Mini Kit and subjected to cDNA preparation with High-Capacity cDNA Reverse Transcription Kit (Thermo Fisher, 4368814). Quantitative PCR was performed using the CFX Connect Real Time System (Bio-Rad) with TaqMan™ Gene Expression Master Mix (Thermo Fisher, 4369016). Primers and probes are purchased from Thermo Fisher.

### Western blotting analysis

Approximately 30 pooled embryos at 24–30 hpf were homogenized in 60 µL protein cocktail (15 µL 4X sample buffer, 10 µL 6X protease inhibitor, 1.5 µL β-Me, and water up to 60 µL). The quantity of the protein loaded onto the western blots was assessed by hybridizing with anti-GAPDH primary antibody (Cell Signaling Technology, 2118) at a dilution of 1:2000. Subsequent SFS-PAGE gels were adjusted based on anti-GAPDH results. p53 was detected by hybridization with anti-p53 primary antibody (GeneTex, 128135) at a dilution of 1:1000, washed and incubated with peroxidase goat anti-rabbit IgG (1:2000; Jackson ImmunoResearch, 111-035-003) and developed with Clarity Western ECL Substrate (Bio-Rad, 1705061). Signal was detected by Bio-Rad ChemiDoc MP system. Western blot images were processed and quantified with Image Lab.

### Establishing tumor cohorts

Our tumor cohorts were established by natural breeding of p53^−/−^ x p53^−/−^ parents. The cohort consisted of 96 fish and was derived from a single set of parents (a single male and female). At 4 months of age, all fish were separated into four tanks of 24 fish each. Adult fish were screened weekly or biweekly for tumors and/or missing/dead fish. Fish that were identified by eye to be tumor burdened were euthanized according to IACUC protocols. Kaplan-Meier analysis was performed using GraphPad Prism 8 software.

### Fluorescence quantification and statistical analysis

GraphPad Prism 8 was used in generation of all graphs and statistical tests. For phenotypic categories and qRT-PCR quantification, overall statistical significance was calculated using an unpaired t-test with error bars indicating SEM. Numbers of embryos and significance values are indicated in the figure legends.

## Supplementary information

supplementary figure legends

Figure S1. Human PUMA and NOXA proteins are conserved in Zebrafish

Figure S2. Quantitative real-time PCR (qRT-PCR) analysis of p53 family members after IR- and drug-induction in wild-type zebrafish embryo

Figure S3. The induction of p73 after IR-irradiation is p53 dependent

Figure S4. Generation and validation of a stable puma/bbc3 mutant in zebrafish

Figure S5. Generation and validation of a stable noxa/pmaip1 mutant in zebrafish

Figure S6. Generation and validation of a stable tp53 mutant in zebrafish

Figure S7. Generation and validation of a tp63 mutant in zebrafish

Figure S8. Generation and validation of a tp73 mutant in zebrafish

Figure S9. Generation and validation of a mdm2 null allele in zebrafish

Figure S10. Quantification of anti-active Caspase-3 staining in wildtype and mutants after 30Gy IR-irradiation

Figure S11. TUNEL staining on 30 hpf (6 h post IR-irradiation) wild-type zebrafish embryos without or with 30Gy IR treatment

Figure S12. Loss of puma but not noxa rescued p53-dependent IR-induced apoptosis by acridine orange (AO) staining

Figure S13. puma is required for BFA-induced apoptosis

Table S1. Primers used for genotyping with HRMs
