# Supplementary material for: Puma, noxa, p53, and p63 differentially mediate stress pathway induced apoptosis
Source: Cell Death Dis. 2021 Jun 30;12(7):659. doi: 10.1038/s41419-021-03902-6 (PMC8245518; doi:10.1038/s41419-021-03902-6)
Supplement: Supplementary file 1 — supplementary figure legends [file 41419_2021_3902_MOESM1_ESM.docx]

**Supplemental Figure Legends:**

**Figure S1. Human PUMA and NOXA proteins are conserved in Zebrafish.** Human and mouse PUMA is 191 and 193 amino acids respectively and both are contained in 4 exons (3 coding exons). The gene coding zebrafish *puma* (181 aa, 4 exons, 3 coding) is consistent with human and mouse PUMA (Figure S1C). Further, the syntenic block containing genes C3H4, SAE1, and PUMA are conserved across human, mouse, and zebrafish. Further the PUMA/NOXA signature QLRXXXDXXN sequence is conserved in zebrafish Puma. Human and mouse NOXA are 54 and 51 amino acids respectively and both are contained in 2 exons. The gene coding zebrafish *noxa* has 2 exons, and codes for a 45 amino acid protein which is consistent with human and mouse NOXA. Further the PUMA/NOXA signature QLRXXXDXXN sequence is conserved in zebrafish *noxa*. However, synteny is not conserved between human and zebrafish NOXA. Together these data support that the Puma and Noxa in zebrafish are homologues of human PUMA and NOXA at the bioinformatic level. **A.** The alignment of amino acid sequences of the BH3 domain for 8 human BH3-only proteins. **B.** The alignment for zebrafish BH3 domain. hHrk is not defined in zebrafish. hBad has two orthologs in zebrafish (bada and badb). The number of their exons, coding exons and protein length are shown. The consensus hydrophobic residue (leucine) and hydrophilic residue (aspartic acid) are indicated with *. **C.** Diagrams of hPuma and zPuma gene. **D.** The alignment of amino acid sequences of the BH3 domain of PUMA in different species. **E.** Diagrams of hNoxa and zNoxa gene. **F.** The alignment of amino acid sequences of the BH3 domain of NOXA across different species. Conserved QLRXXXDXXN of PUMA and NOXA are labelled with dark orange.

**Figure S2. Quantitative real-time PCR (qRT-PCR) analysis of p53 family members after IR- and drug-induction in wild-type zebrafish embryos.** 24hpf zebrafish embryos were treated with (**A**) 30Gy IR-irradiation, (**B**) 5μM Thapsigargin (Thaps.), and (**C**) 3.3μM Phorbol 12-myristate 13-acetate (PMA); and qRT-PCR was performed at 6h (**A**) or 4h (**B and C**) after treatment. Two commercial TaqMan probes of *p53* and *p63* were performed to validate the relative expression results. Expression levels were normalized to *actb1*. n=9 (**A and B**) and n=7 (**C**) from ~30 pooled embryos per sample. Bars represent mean ± SEM. **, p < 0.01; ***, p < 0.001; ****, p < 0.0001.

**Figure S3. The induction of *p73* after IR-irradiation is *p53* dependent.** Quantitative real-time PCR (qRT-PCR) analysis of *p53* and *p73* after 6-hour 30Gy IR-irradiation in wild-type and *tp53*-null zebrafish embryos. Two commercial TaqMan probes of *p53* were performed to validate the relative expression results. Expression levels were normalized to GAPDH. n=6 (both wild-type and *tp53*^-/-^ groups) from ~30 pooled embryos per sample. Bars represent mean ± SEM. ***, p < 0.001; ****, p < 0.0001.

**Figure S4. Generation and validation of a stable *puma/bbc3* mutant in zebrafish. A.** Diagram of the target site in zebrafish *bbc3* genome. *bbc3* endogenous locus, TALEN target site in exon 2 of the *bbc3* gene (arrow), TALEN sequences (red). We identified multiple alleles however only propagated a 2bp deletion that results in a frame shift at codon 69. We predict that this truncation will be out-of-function since it lacks the essential BH3 domain. **B.** Sequence alignment between *bbc3* wildtype and mutant (Δ2) alleles. Red indicates the two nucleotides deleted in mutant allele. Purple indicates the out-in-frame nucleotides. **C.** The wild-type and truncated Puma protein. Red indicates the out-of-frame amino acid sequence in mutant allele. One concern when making indel alleles is that the mutation induces an alternative transcript that could potentially make a functional protein. To address this, we performed RT-PCR of mRNA (primers flanking 5’ and 3’ UTR) from wild-type and homozygous mutant embryos and did not find any alternative transcripts. **D.** RT-PCR amplification of the *bbc3* wild-type (+/+) and homozygous (-/- = Δ2/Δ2) transcripts. Often premature stop codons will induce nonsense mediated decay (NMD) of the mutant mRNA transcript. To address if our mutant allele induced NMD, we performed sequencing of the RT-PCR product from heterozygous-null tail mRNA. If there is compete NMD of the mutant allele, we would expect to only see the wild-type sequence on the chromatogram, however if there is no NMD, we would observe both sequences in the chromatogram. **E.** Nonsense mediated decay (NMD) sequence chromatogram of *bbc3* heterozygous zebrafish. For the *puma* Δ2 allele (hence forth referred to as the *bbc3*^-/-^allele), the mutant sequence is present at the same levels as the wild-type sequence (Figure S2E) suggesting no NMD of the mutant mRNA. **F.** Lateral views of a female (above) and male (below) *bbc3*^-/-^ adult zebrafish. Homozygous *puma* nulls are viable, fertile, and free of tumors

**Figure S5. Generation and validation of a stable *noxa/pmaip1* mutant in zebrafish. A.** Diagram of the target site in the zebrafish *pmaip1* genome. *pmaip1* endogenous locus, TALEN target site in exon 2 (codon 10) of the *pmaip1* gene (arrow), TALEN sequences (red). We propagated an out of frame allele (Δ 5+4) that deletes 5 nucleotides and inserts 4 nucleotides **B.** Sequence alignment between *pmaip1* wildtype and stable mutant (Δ5+4). Red indicates the five nucleotides deleted and underline labels the four nucleotides inserted in mutant allele. Purple indicates the out-in-frame nucleotides. This mutation results in a frameshift at codon 11 and truncation that is 5’ of the BH3 domain of wild-type *noxa* and is predicted to result in a non-functional protein. **C.** The wild-type and truncated Noxa protein. Red indicates the out-of-frame amino acid sequence in mutant allele. We did not observe any alternative transcripts or NMD in the mutant allele **D.** RT-PCR amplification of *pmaip1* wild-type (+/+) and homozygous (-/- = Δ5+4/Δ5+4) transcripts (the lower band). The upper band indicates the products include intron 1. **E.** NMD sequence chromatogram of *pmaip1* heterozygous zebrafish. **F.** Lateral views of a female (above) and a male (below) *pmaip1*^-/-^ adult zebrafish. Homozygous *noxa* nulls (hence forth referred to as pmaip1^-/-^) are viable and fertile without tumor formation.

**Figure S6. Generation and validation of a stable *tp53* mutant in zebrafish.** Previous zebrafish *p53* mutant models were missense mutations that produce a protein, which could have dominant negative or gain of function activity (44). To generate a true *p53* null allele, we designed a CRISPR guide that targets codon 57 in exon 4 of zebrafish *p53.* **A.** Diagram of the target site in the zebrafish *tp53* genome. *tp53* endogenous locus, gRNA target site in exon 4 of the *tp53* gene (arrow), PAM motif (red). The propagated out-of-frame allele has an insertion of 2 bp (+2) at codon 58, resulting in a truncation within the transactivation domain, before the DNA binding domain and oligomerization domain. **B.** Sequence alignment between *tp53* wildtype and stable mutant (+2). the two nucleotides inserted are underlined and purple indicates the out-in-frame nucleotides. **C.** The wild-type and truncated p53 protein. Red indicates the out-of-frame amino acid sequence in mutant allele. This allele does not result in an alternative spliced product, nor undergoes NMD, and no protein is detected by western blot after IR treatment **D.** PCR amplification of *tp53* wild-type (+/+) and homozygous (-/- = +2/+2) transcripts. **E.** NMD sequence chromatogram of *tp53* heterozygous zebrafish. **F.** p53 protein expression level w/ or w/o IR-irradiation. Western blot analysis was performed using wild-type or *tp53*^-/-^ zebrafish embryos. The embryos were treated with 30Gy IR-irradiation at 24hpf and approximately 30 embryos were homogenized in protein cocktail at 6h post induction. Homozygous *p53* nulls (hence forth referred to as *p53^-/-^)* are viable and fertile. Aged homozygous animals develop tumors at an early age consistent with other *p53* mutant alleles in zebrafish and mouse (33, 34, 44, 62). Interestingly, the timing of tumor onset is faster than our published *p53* missense allele (T_50_ = 247 vs 320, Fig S4G), suggesting the previous missense allele may be hypomorphic. **G.** Kaplan-Meier tumor-free survival of *tp53* ^-/-^ (blue curve; T50=247 days) zebrafish compared with *tp53* ^M/M^ (I166T, green curve) and wildtype allele (orange curve). Long-rank statistic test was done. ****, p value between *tp53* ^-/-^ and *tp53* ^M/M^ < 0.0001 and p value between *tp53*^-/-^ and *tp53*^+/+^ < 0.0001. **H.** Lateral views of *tp53*^-/-^ fish with skin, eye, flank, or abdominal tumor.

**Figure S7. Generation and validation of a *tp63* mutant in zebrafish.** *p63* has two main alternative transcripts, the TA and the ΔN. To generate a null in both transcripts we targeted codon 263 in the TA and codon 170 of the ΔN, with a zinc finger nuclease **A.** Diagram of the target site in zebrafish *tp63* genome. *tp63* endogenous locus, Zinc finger target site of the *tp63* gene (arrow), zinc finger sequences (red). We propagated a line with a 4 bp insertion resulting in a premature stop codon. **B.** Sequence alignment between *tp63* wildtype and mutant (+4). Underline labels the four nucleotides inserted in mutant allele and purple indicates the out-in-frame nucleotides. **C.** This truncation disrupts the DNA binding domain and prevents the ability to transcriptionally induce target genes. The wild-type and truncated p63 protein. Red indicates the inserted stop codon in mutant allele. The mutant allele undergoes strong NMD suggesting none of the protein will be generated. **D.** genomic DNA (gDNA) and NMD (cDNA) sequence chromatogram of *tp63* heterozygous zebrafish. Immunohistochemistry of *p63* null embryos reveals no staining in homozygous mutant embryos, while wild-type siblings had the expected nuclear staining on the epithelium. **E.** whole-embryonic anti-p63 staining on 3dpf *tp63*^+/+^ and *tp63*^-/-^ (-/- = +4/+4) zebrafish embryos. p63 (green) and DAPI (Blue). Scale bar, 1000μM. Homozygous p63 null are non-viable and display the first phenotypic deformities at 2.5dpf, the majority displaying phenotypes at 3dpf. Additionally, there are no *p63* nulls viable at 5dpf. **F.** Gross images of 3dpf *tp63*^+/+^ and *tp63*^-/-^ zebrafish embryos. The number and timeline of *tp63*^-/-^ embryos shown abnormal phenotype (as figure above) in three different clutches. Mendelian ratio from three different clutches and genotyping result of rest normal embryos at 120hpf (5dpf) shown. Note there is no morphological abnormalities at 24hpf. **G.** Gross images of 24hpf *tp63*^+/+^ and *tp63*^-/-^ zebrafish embryos. Scale bar, 1000μM.

**Figure S8. Generation and validation of a *tp73* mutant in zebrafish.** While human p73 has two alternative transcripts, the TA and the ΔN, in zebrafish only the TA version has been observed. To generate a p73 null allele, we targeted codon 112 in exon 3 with a CRISPR guide **A.** Diagram of the target site in zebrafish *tp73* genome. *tp73* endogenous locus, gRNA target site of the *tp73* gene (arrow), PAM motif (red). We propagated a Δ8 allele which generates a frame shift at codon 112. **B.** Sequence alignment between *tp73* wildtype and mutant (Δ8). Red indicates the eight nucleotides deleted in mutant allele and purple indicates the out-in-frame nucleotides. We predict this allele will be a null allele since it truncates the protein prior to the essential DNA binding, oligomerization and SAM domains. **C.** The wild-type and truncated p73 protein. Red indicates the out-of-frame amino acid sequence in mutant allele. Further, the p73 null allele undergoes NMD, thereby not producing any protein. **D.** gRNA and NMD sequence chromatogram of *tp73* heterozygous zebrafish. The homozygous null zebrafish are viable and fertile. **E.** Lateral views of a female (above) and male (below) *tp73*^-/-^ (-/- = Δ8/Δ8) adult zebrafish.

**Figure S9. Generation and validation of a *mdm2* null allele in zebrafish.** We have generated an *mdm2* null allele by targeting a pair of TALENs to codon 135 in exon 7 of zebrafish mdm2 **A.** Diagram of the target site in the zebrafish *mdm2* genome. *mdm2* endogenous locus, TALEN target site in exon 7 of the *mdm2* gene (arrow), TALEN sequences (red). The propagated allele has a deletion of 7 bp resulting in a truncation at codon 136 (hence forth referred to as *mdm2^-/-^*). **B.** Sequence alignment between *mdm2* wildtype and stable mutant (Δ7). Red indicates the seven nucleotides deleted in mutant allele. Purple indicates the out-in-frame nucleotides. **C.** The wild-type and truncated Mdm2 protein. Red indicates the out-of-frame amino acid sequence in mutant allele. No NMD was detected in this allele. **D.** NMD sequence chromatogram of *mdm2* heterozygous zebrafish. Progenies from heterozygous crosses resulted in a quarter of the clutch undergoing abnormal development as early as 15hpf. Abnormal embryos were homozygous *mdm2* mutant, while no homozygous mutant embryos were identified in the “normal” phenotypic embryos **E.** Gross images of 15hpf, 18hpf and 21hpf *mdm2*^+/+^ and *mdm2*^-/-^ (-/- = Δ7/Δ7) zebrafish embryos. Scale bar, 500μM. Mendelian ratio from three different clutches and genotyping result of abnormal and normal embryos at 15hpf are shown. To determine if this lethality was p53 dependent, we crossed the *mdm2* null into the *p53* null background. We obtained viable double null embryos and adults. **F.** Loss of p53 rescued *mdm2*-null induced early embryonic lethality. 24hpf *mdm2*^-/-^ and *mdm2*^-/-^ *tp53*^-/-^ zebrafish embryos. Scale bar, 500μM. **G**. Lateral views of a female (above) and a male (below) *mdm2*^-/-^ adult zebrafish. These data indicate that the mdm2 regulation of p53 is conserved in zebrafish and the mdm2 null embryos represents uncontrolled activation of p53.

**Figure S10. Quantification of anti-active Caspase 3 staining in wildtype and mutants after 30Gy IR-irradiation.** Anti-active Caspase-3 staining on 30hpf (6 hours post IR-irradiation) wild-type, *pmaip1*^-/-^, *tp53*^-/-^, *tp63*^-/-^, *tp73*^-/-^ and *bbc3*^-/-^ embryos. Mean fluorescence intensity (MFI) of the head regions of IR-treated embryos were quantified. N>20 for wildtype and each mutant from three indepdent experiments.

**Figure S11. TUNEL staining on 30hpf (6 hours post IR-irradiation) wild-type zebrafish embryos without or with 30Gy IR treatment. A.** Representative figures showing the apoptotic phenotype at the head and neural tube region. Scale bar, 1000μM. **B.** Quantification of fluorescence intensity of head and neural tube of non-IR and 30Gy IR-treated embryos with TUNEL staining. Each dot represents MFI of the head and neural tube region of individual embryos from two independent experiments. Bars represent mean ± SEM. ****, p < 0.0001.

**Figure S12. Loss of *puma* but not *noxa* rescued p53-dependent IR-induced apoptosis by acridine orange (AO) staining. A.** AO staining on 30hpf zebrafish w/ different IR-irradiation. 24hpf zebrafish embryos were treated with 0Gy, 15Gy, 30Gy, 45Gy, 60Gy and 100Gy IR-irradiation and were fixed at 6h after IR-induction for wild-type, *tp53*^-/-^, *bbc3*^-/-^ and *pmaip1*^-/-^ zebrafish lines. Scale bar, 1000μM.

**Figure S13. *puma* is required for BFA-induced apoptosis A.** anti-active Caspase-3 staining on 30hpf (6 hours post treatment) wildtype zebrafish embryos with DMSO or DMSO plus 10μM BFA treatment. Representative figures showing the degree of phenotypic severity. **B.** Quantification of mean fluorescent intensity (MFI) in tail region in DMSO treated and BFA treated embryo in mild and severe categories. Each dot represents mean MFI of individual embryos. Bars represent mean ± SEM. ****, p < 0.0001. **C.** qRT-PCR analysis of pro-apoptotic markers 6 hours post DMSO or DMSO plus 10μM BFA treatment. n=6 from at least 30 pooled embryos per sample. Bars represent mean ± SEM. *, p < 0.05; **, p < 0.01. **D.** Percentage of phenotypic categories in wildtype and *bbc3*^-/-^ zebrafish embryos after DMSO or DMSO plus 10μM BFA treatment. n=4 from pooled embryos per sample. The total number of BFA-treated embryos: wildtype or *bbc3*^-/-^ > 400. Bars represent mean ± SEM. ****, p < 0.0001.
