# Supplementary figures and images for: Puma, noxa, p53, and p63 differentially mediate stress pathway induced apoptosis
Source: Cell Death Dis. 2021 Jun 30;12(7):659. doi: 10.1038/s41419-021-03902-6 (PMC8245518; doi:10.1038/s41419-021-03902-6)

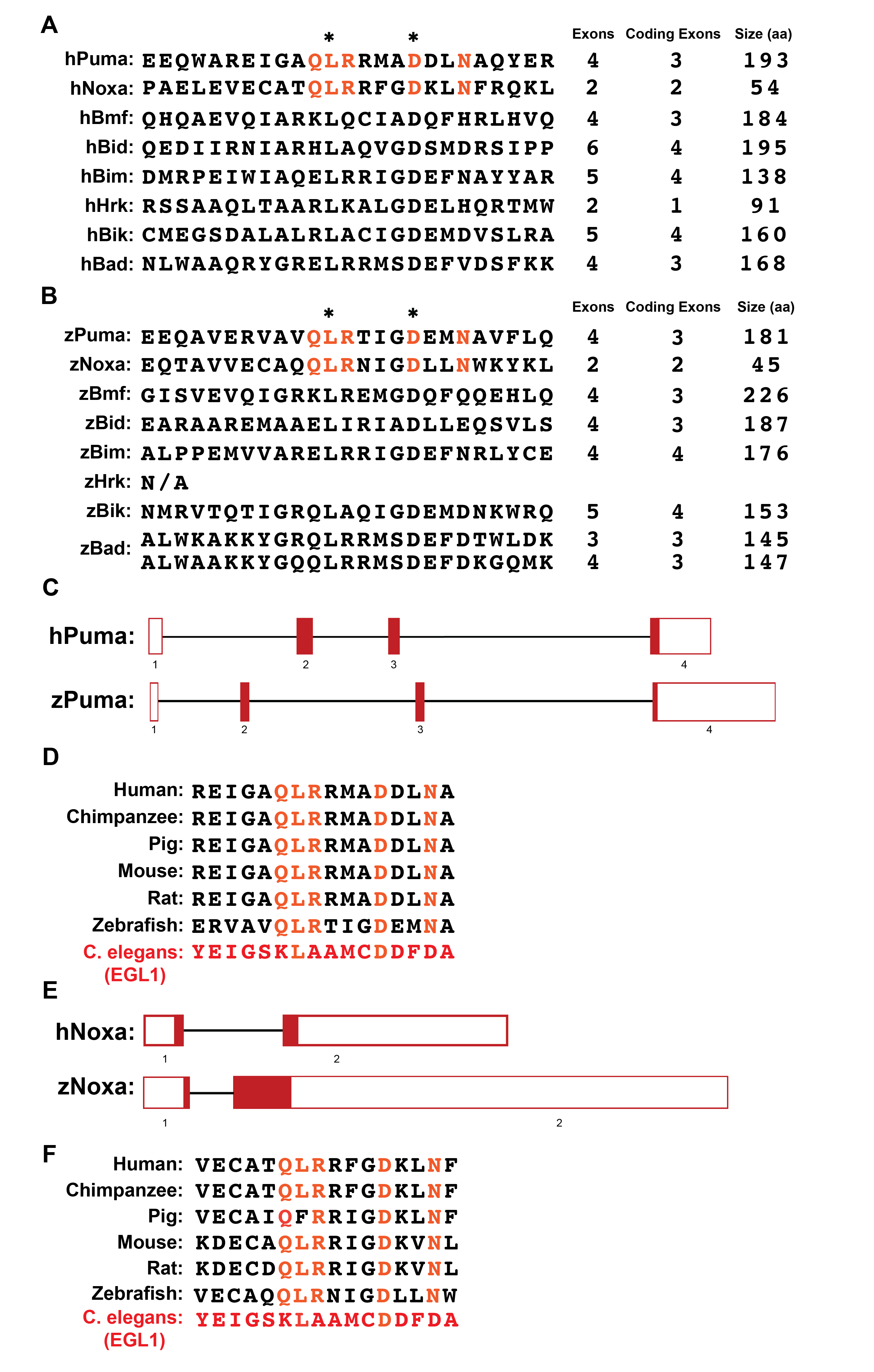

Supplement: Supplementary file 2 — Figure S1. Human PUMA and NOXA proteins are conserved in Zebrafish [file 41419_2021_3902_MOESM2_ESM.png]

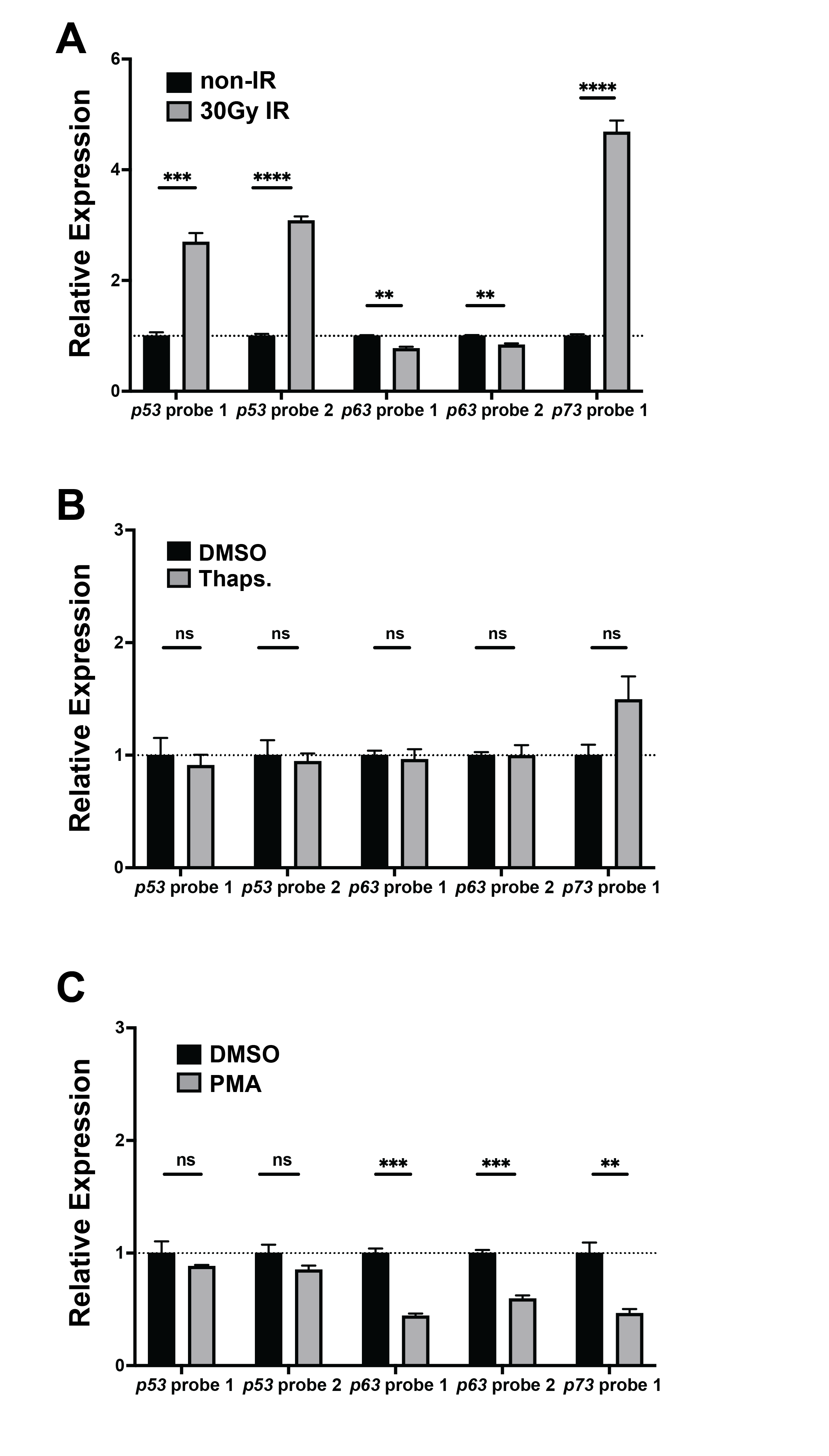

Supplement: Supplementary file 3 — Figure S2. Quantitative real-time PCR (qRT-PCR) analysis of p53 family members after IR- and drug-induction in wild-type zebrafish embryo [file 41419_2021_3902_MOESM3_ESM.png]

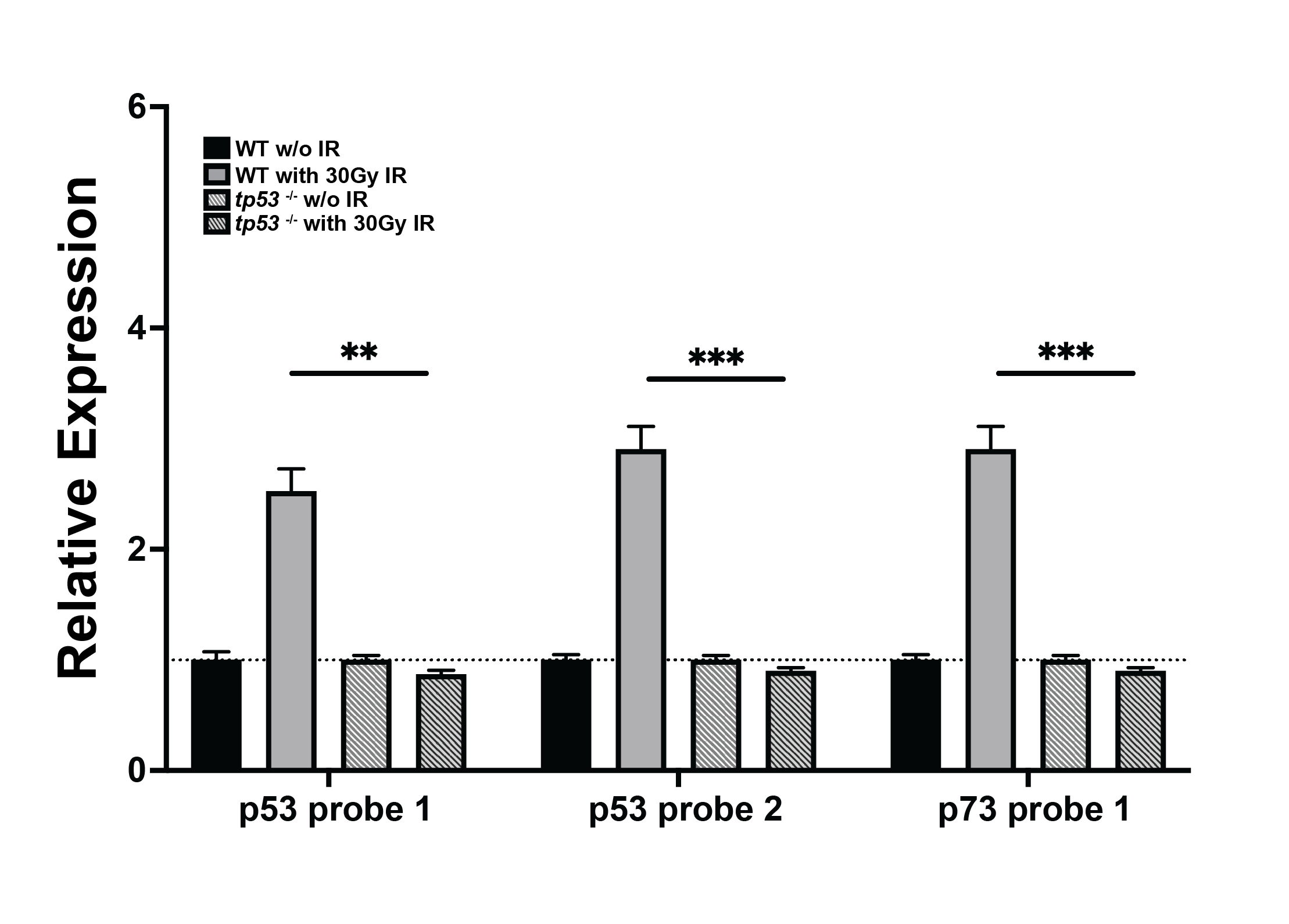

Supplement: Supplementary file 4 — Figure S3. The induction of p73 after IR-irradiation is p53 dependent [file 41419_2021_3902_MOESM4_ESM.png]

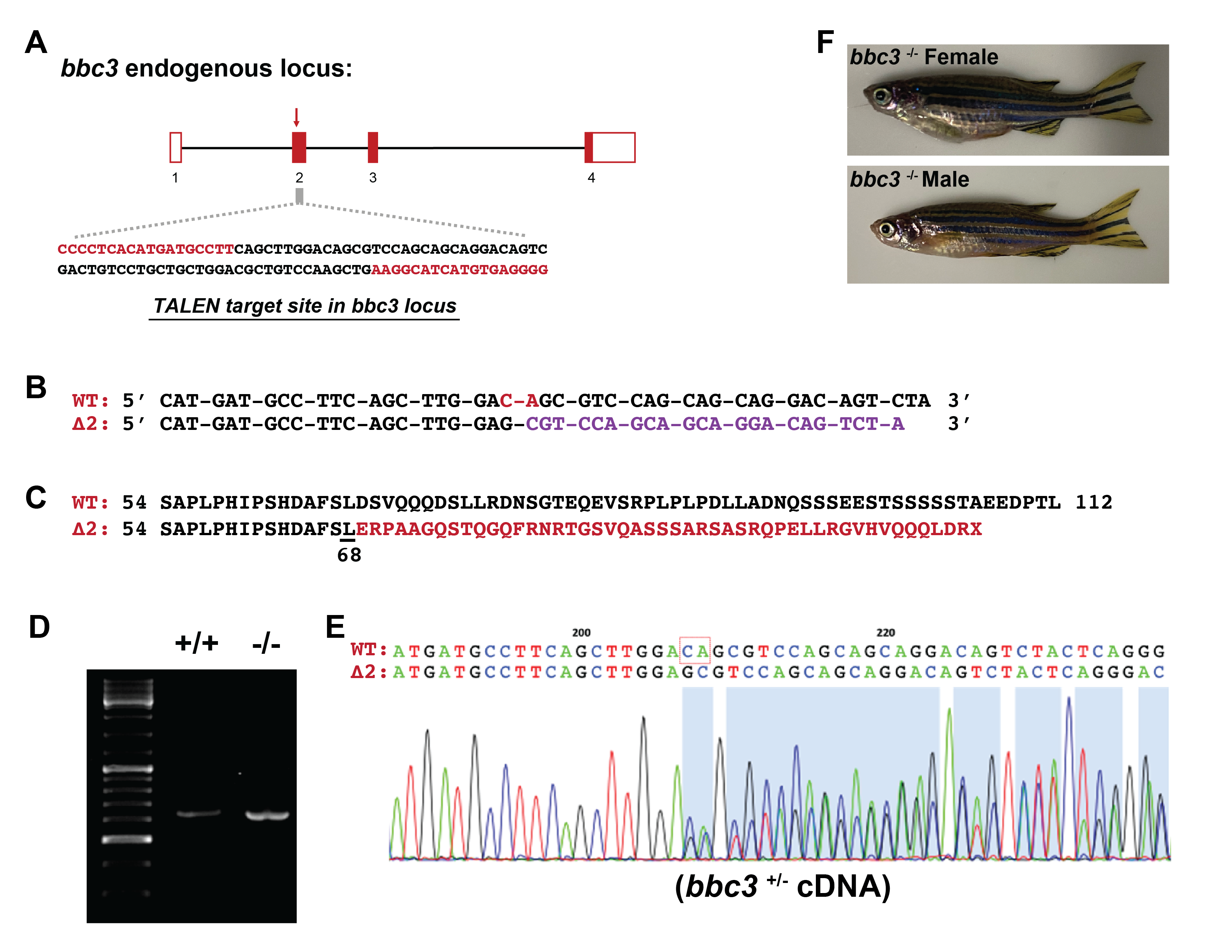

Supplement: Supplementary file 5 — Figure S4. Generation and validation of a stable puma/bbc3 mutant in zebrafish [file 41419_2021_3902_MOESM5_ESM.png]

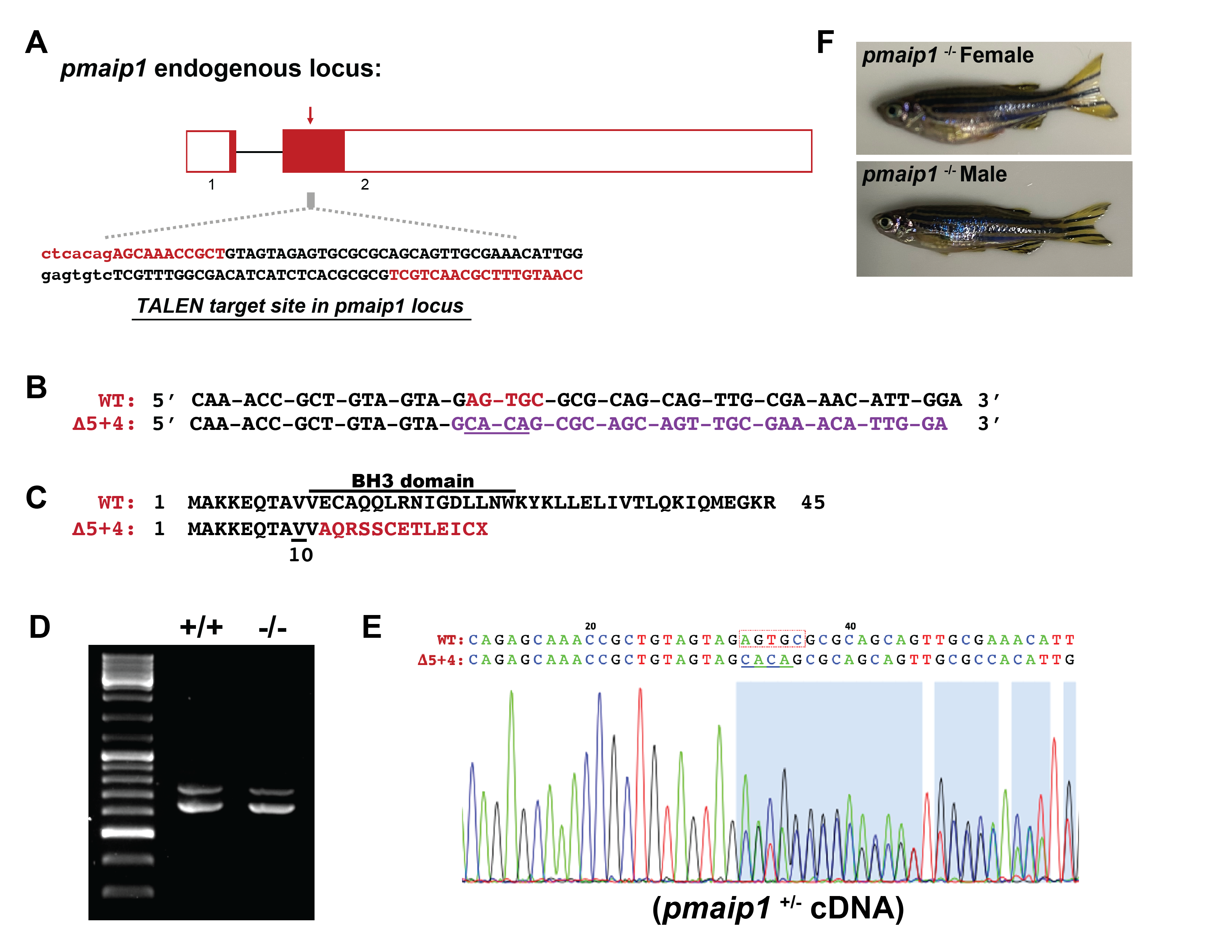

Supplement: Supplementary file 6 — Figure S5. Generation and validation of a stable noxa/pmaip1 mutant in zebrafish [file 41419_2021_3902_MOESM6_ESM.png]

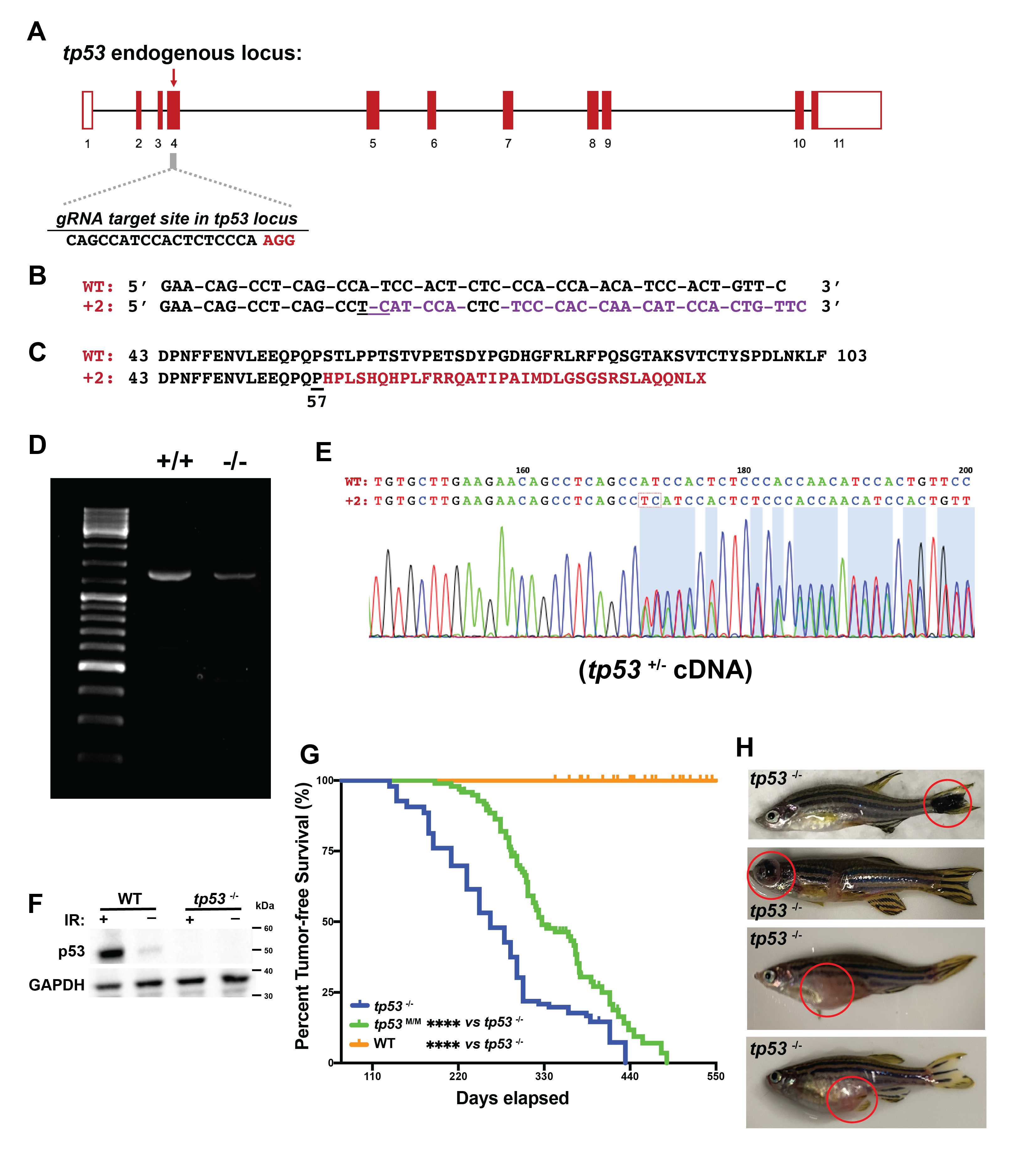

Supplement: Supplementary file 7 — Figure S6. Generation and validation of a stable tp53 mutant in zebrafish [file 41419_2021_3902_MOESM7_ESM.png]

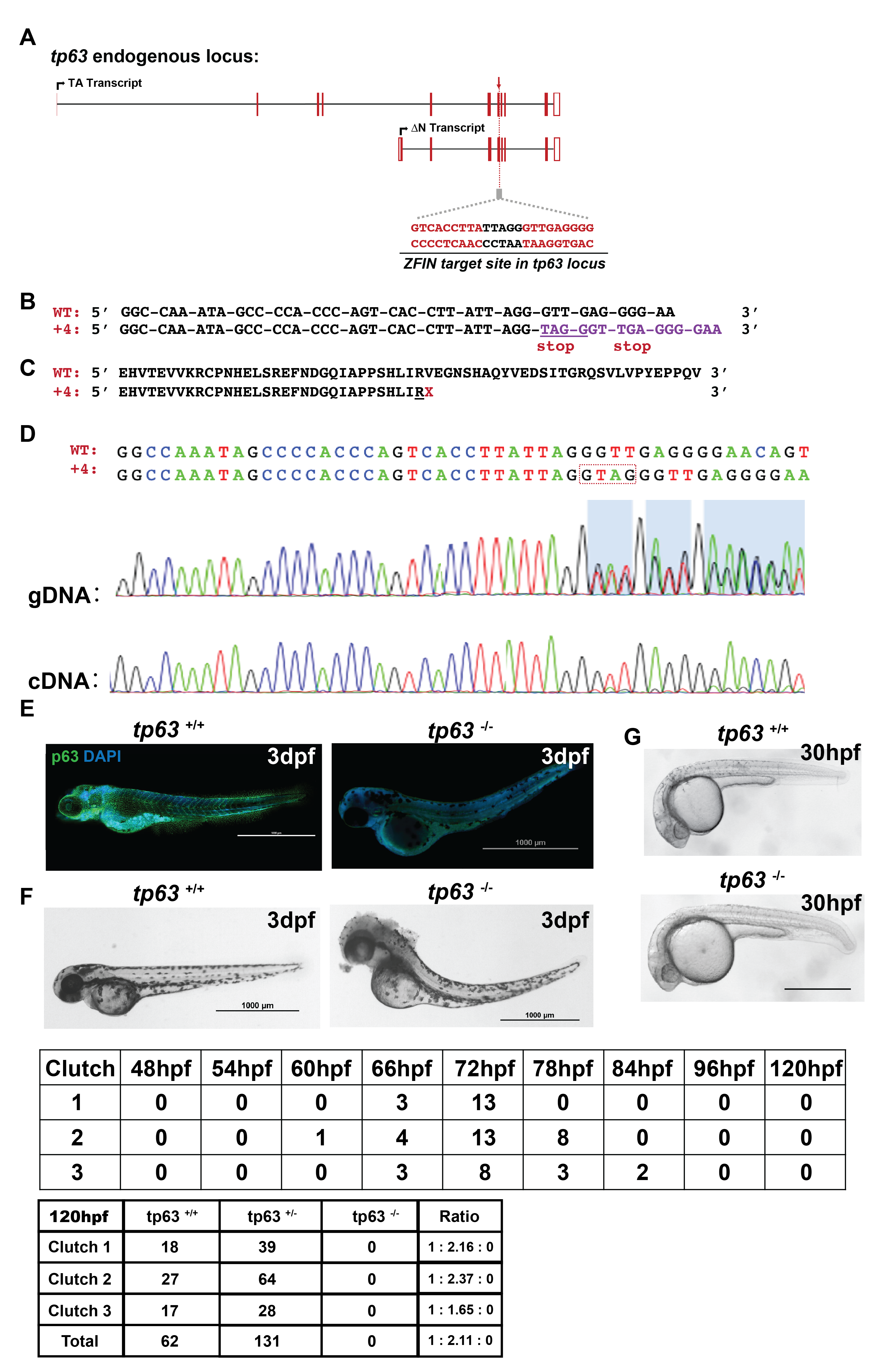

Supplement: Supplementary file 8 — Figure S7. Generation and validation of a tp63 mutant in zebrafish [file 41419_2021_3902_MOESM8_ESM.png]

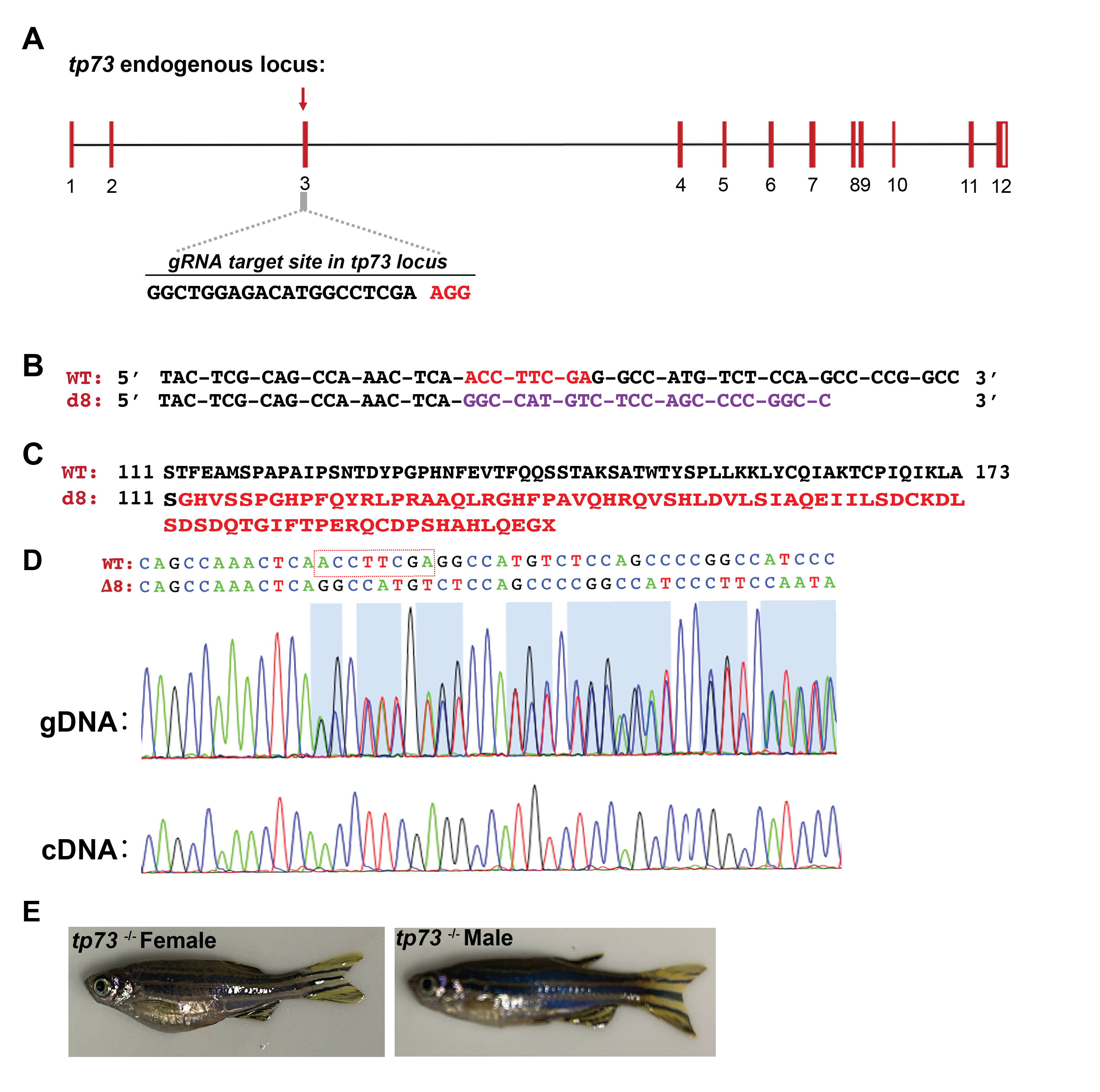

Supplement: Supplementary file 9 — Figure S8. Generation and validation of a tp73 mutant in zebrafish [file 41419_2021_3902_MOESM9_ESM.png]

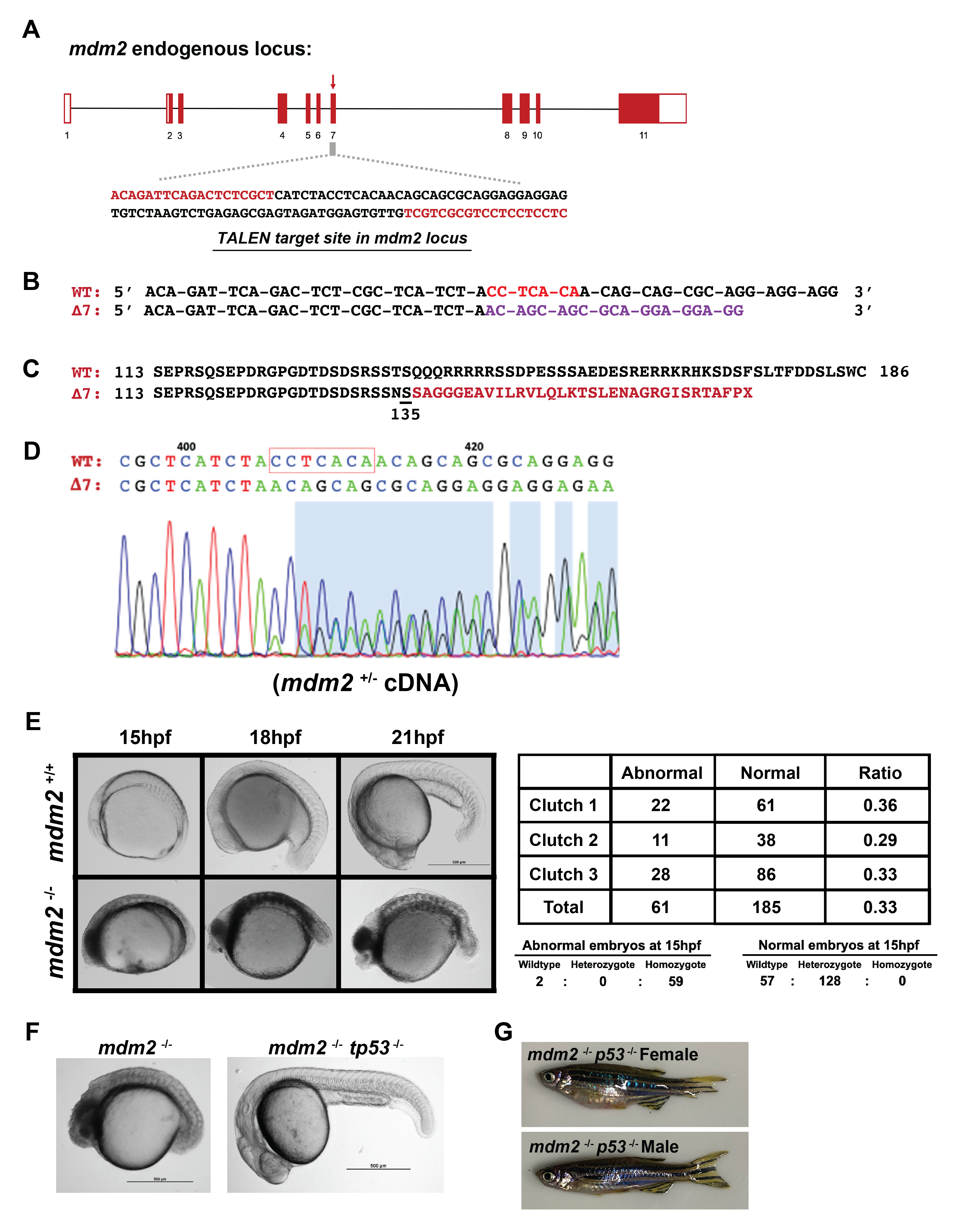

Supplement: Supplementary file 10 — Figure S9. Generation and validation of a mdm2 null allele in zebrafish [file 41419_2021_3902_MOESM10_ESM.png]

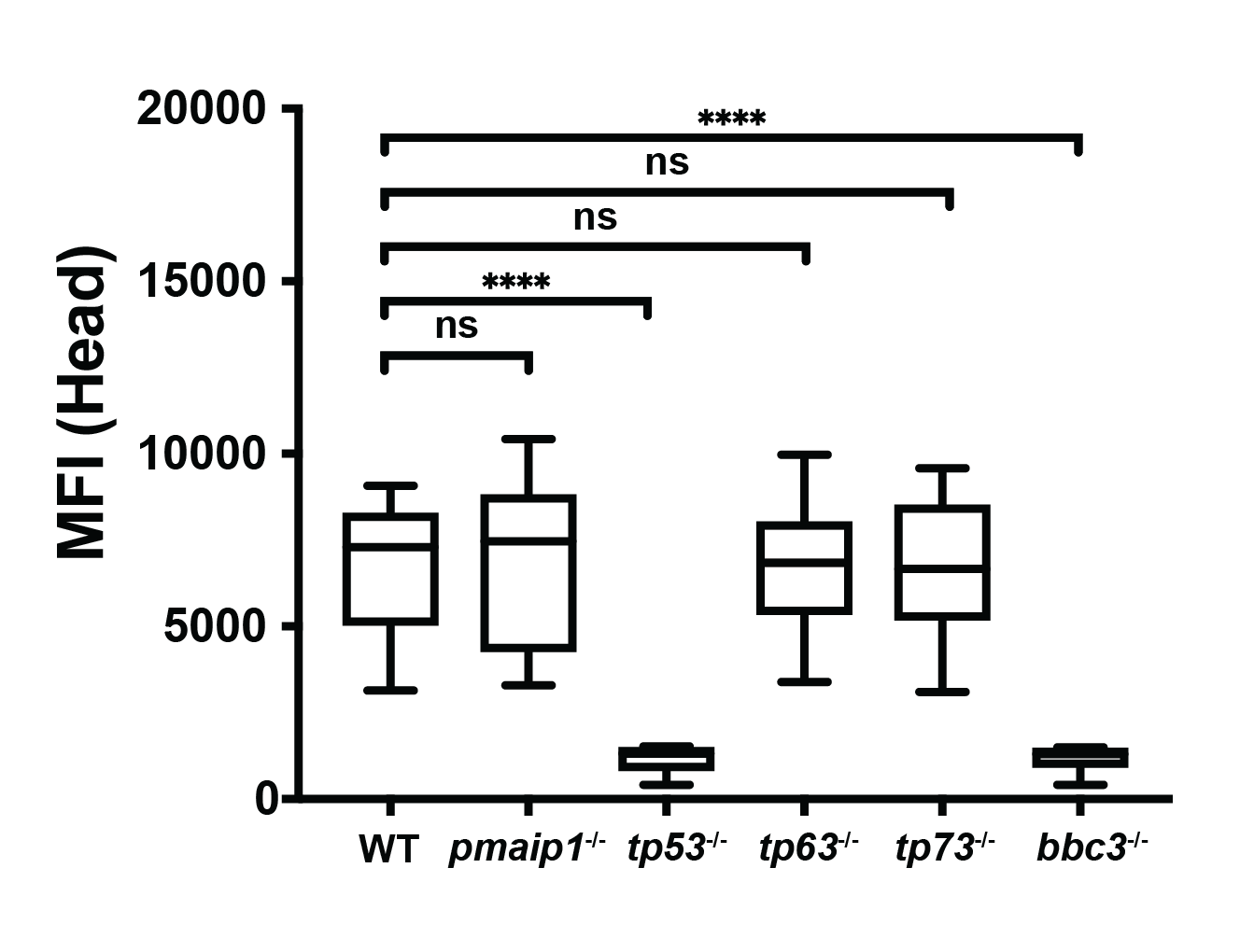

Supplement: Supplementary file 11 — Figure S10. Quantification of anti-active Caspase-3 staining in wildtype and mutants after 30Gy IR-irradiation [file 41419_2021_3902_MOESM11_ESM.png]

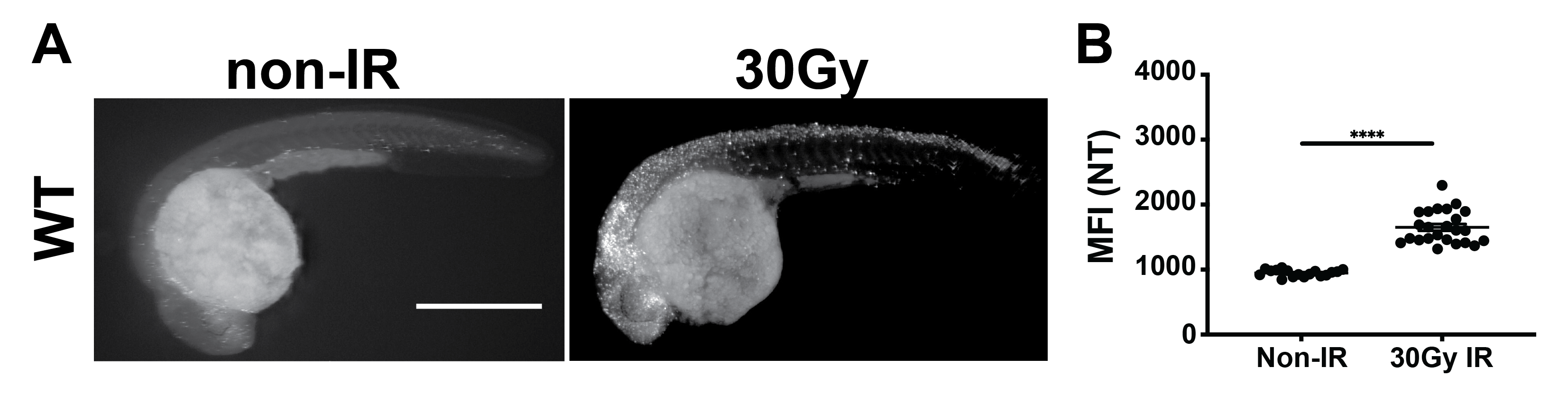

Supplement: Supplementary file 12 — Figure S11. TUNEL staining on 30 hpf (6 h post IR-irradiation) wild-type zebrafish embryos without or with 30Gy IR treatment [file 41419_2021_3902_MOESM12_ESM.png]

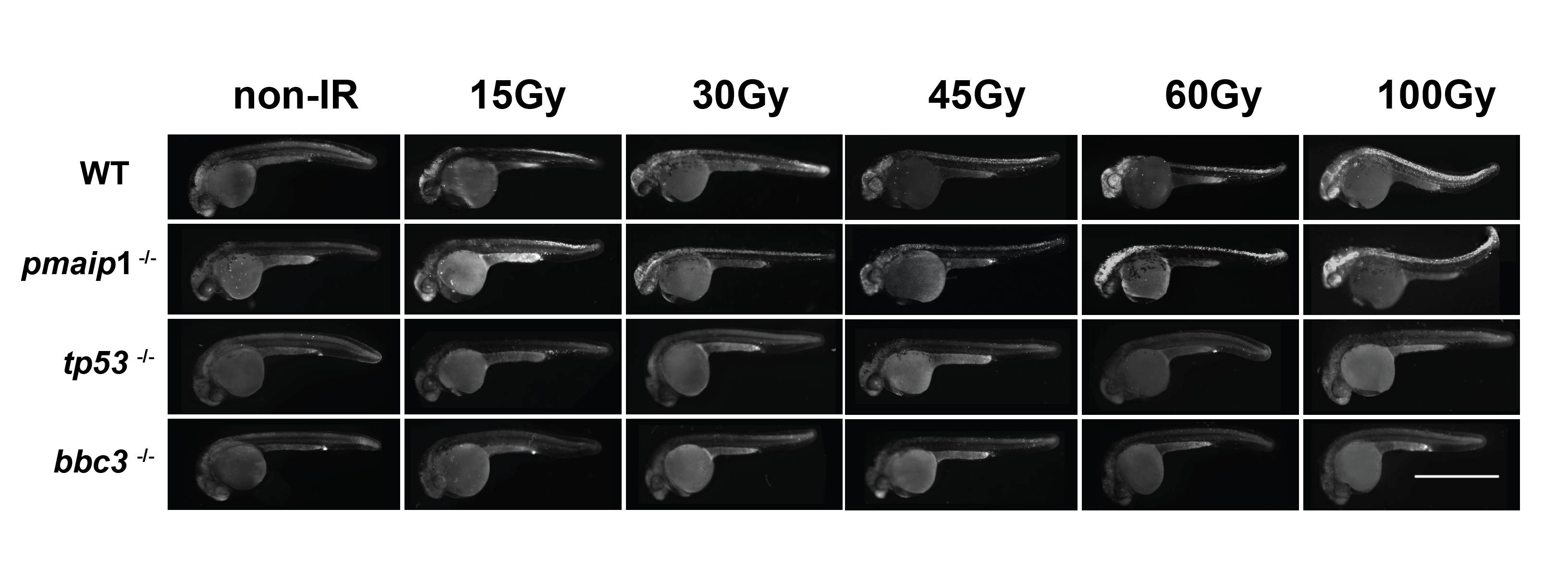

Supplement: Supplementary file 13 — Figure S12. Loss of puma but not noxa rescued p53-dependent IR-induced apoptosis by acridine orange (AO) staining [file 41419_2021_3902_MOESM13_ESM.png]

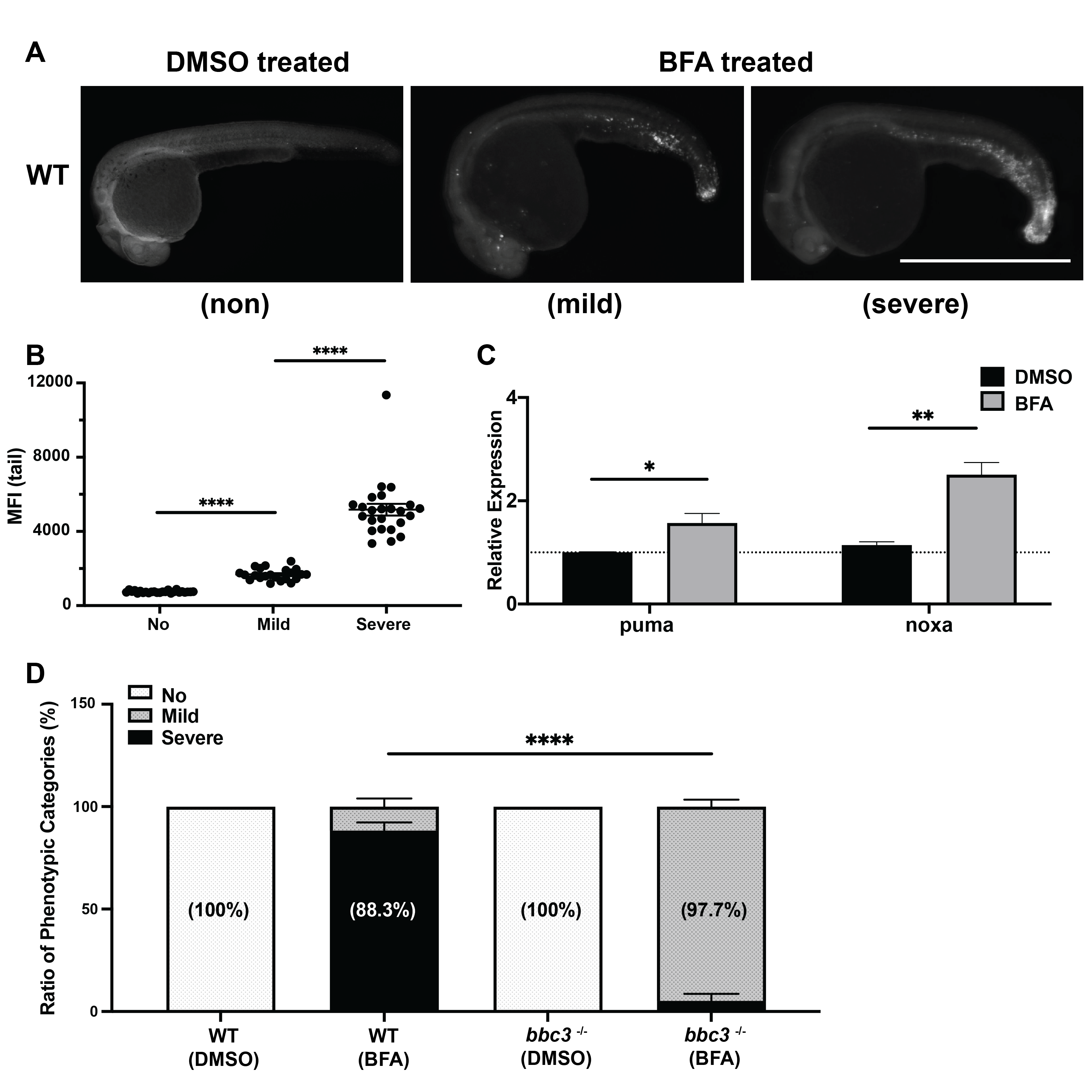

Supplement: Supplementary file 14 — Figure S13. puma is required for BFA-induced apoptosis [file 41419_2021_3902_MOESM14_ESM.png]

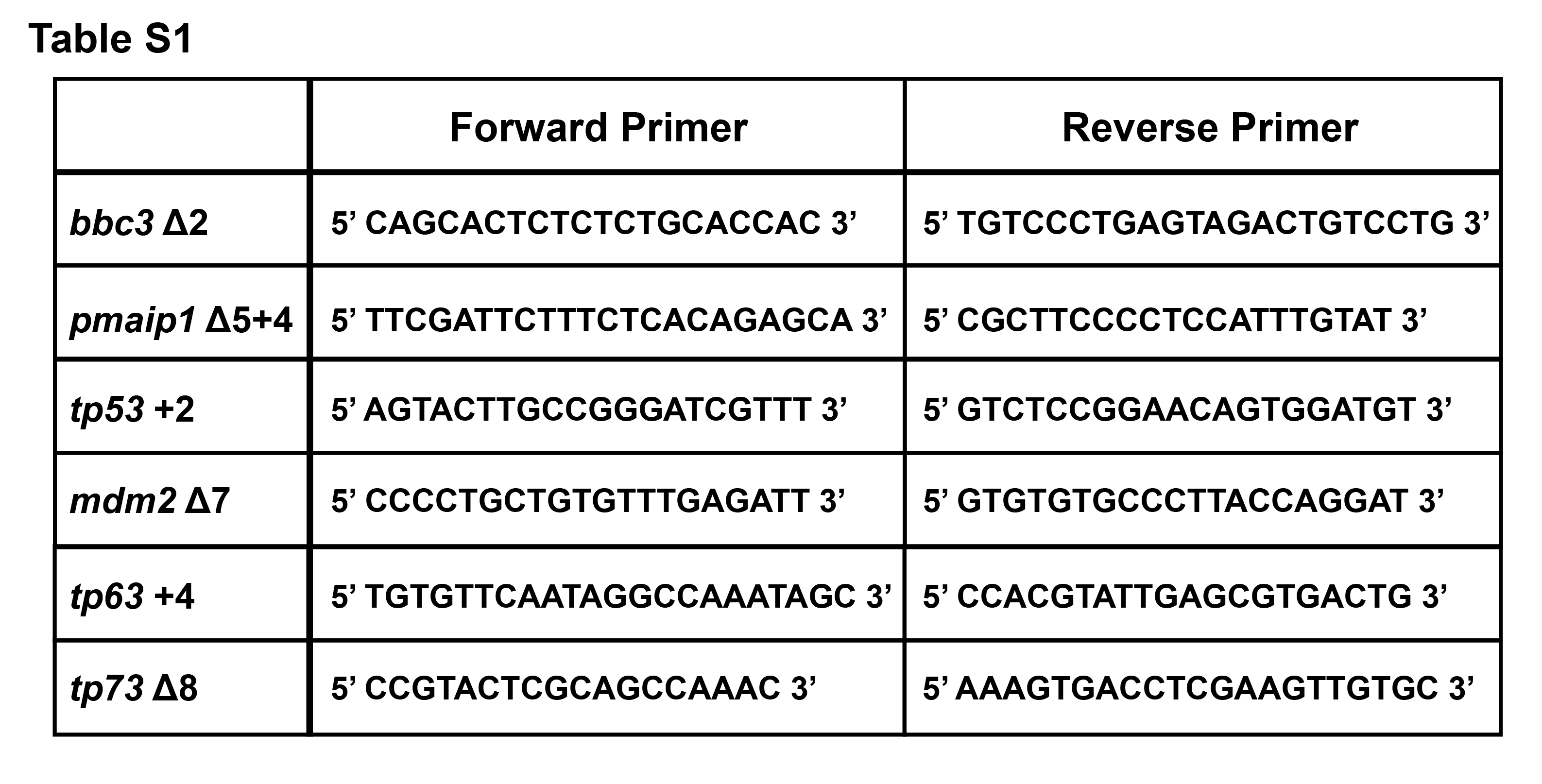

Supplement: Supplementary file 15 — Table S1. Primers used for genotyping with HRMs [file 41419_2021_3902_MOESM15_ESM.png]
